# Building long-term empathy: A large-scale comparison of traditional and virtual reality perspective-taking

**DOI:** 10.1371/journal.pone.0204494

**Published:** 2018-10-17

**Authors:** Fernanda Herrera, Jeremy Bailenson, Erika Weisz, Elise Ogle, Jamil Zaki

**Affiliations:** 1 Department of Communication, Stanford University, Stanford, California, United States of America; 2 Department of Psychology, Stanford University, Stanford, California, United States of America; University of Melbourne, AUSTRALIA

## Abstract

Virtual Reality (VR) has been increasingly referred to as the “ultimate empathy machine” since it allows users to experience any situation from any point of view. However, empirical evidence supporting the claim that VR is a more effective method of eliciting empathy than traditional perspective-taking is limited. Two experiments were conducted in order to compare the short and long-term effects of a traditional perspective-taking task and a VR perspective-taking task (Study 1), and to explore the role of technological immersion when it comes to different types of mediated perspective-taking tasks (Study 2). Results of Study 1 show that over the course of eight weeks participants in both conditions reported feeling empathetic and connected to the homeless at similar rates, however, participants who became homeless in VR had more positive, longer-lasting attitudes toward the homeless and signed a petition supporting the homeless at a significantly higher rate than participants who performed a traditional perspective-taking task. Study 2 compared three different types of perspective-taking tasks with different levels of immersion (traditional vs. desktop computer vs. VR) and a control condition (where participants received fact-driven information about the homeless). Results show that participants who performed any type of perspective-taking task reported feeling more empathetic and connected to the homeless than the participants who only received information. Replicating the results from Study 1, there was no difference in self-report measures for any of the perspective-taking conditions, however, a significantly higher number of participants in the VR condition signed a petition supporting affordable housing for the homeless compared to the traditional and less immersive conditions. We discuss the theoretical and practical implications of these findings.

## Introduction

Empathy, the ability to share and understand someone else’s emotions, is an imperative component of successful social interactions [1]. Empathy has been shown to increase understanding and motivate prosocial behaviors [2–5]. Given its importance and positive effects on intergroup social interactions, researchers, artists, and tech companies have tried to find novel ways to increase empathy via different types of empathy interventions.

In 2015, virtual reality (VR) was described as the “ultimate empathy machine” since it allows people to viscerally experience anything from another person’s point of view [6]. VR experiences, or immersive virtual environments (IVEs), are computer generated, 3D environments where people can move around freely and interact with their surroundings. IVEs replace the user’s perceptual input of the real world with perceptual input from a virtual world, and make users feel like they are actually inside a virtual environment. De la Peña and colleagues [7] argue that VR’s ability to elicit *presence*, the user’s subjective feeling of being inside an IVE, allows users to more deeply understand perspectives other than their own. Since then, companies and institutions like Facebook, HTC, and the United Nations have allocated millions of dollars toward programs such as Oculus’s “VR for Good” and HTC’s “VR for Impact” [8–9], which try to use VR to promote empathy and social welfare.

Interest in VR as an empathy tool has led to the increased production of IVEs designed with the sole purpose of increasing empathy. These experiences place users in novel environments, showing them what it would be like to experience a specific situation from someone else’s perspective. Extensive research shows that taking the perspective of someone else (i.e., imagining what it would be like to be someone else) can be an effective method of promoting empathy and motivating prosocial behaviors [2–5, 10–12]. However, there is a need for more empirical evidence validating perspective-taking in VR as an effective way to promote empathy, especially now that multiple empathy-driven IVEs are publicly available online, and past research demonstrates that traditional perspective-taking does not always lead to positive outcomes [13]. Thus, the current investigation focuses on 1) comparing the short and long-term effects of a traditional perspective-taking task against a VR perspective-taking task, and 2) exploring the technological and psychological mechanisms that make perspective-taking an effective method of promoting empathy and prosocial behaviors.

### Positive and negative effects of perspective-taking

Traditional perspective-taking tasks (i.e., where participants are asked to imagine what it would be like to be someone else under specific circumstances), have been used extensively in the literature, and can effectively increase the empathy a perceiver feels for a specific social target. These tasks can be particularly powerful since they often result in feelings of inclusion and self-other merging [10], reduce prejudice and attenuate negative stereotypes [14], can help create and maintain social bonds [15], facilitate social interactions [16], and motivate altruistic helping behaviors [12]. In addition, perspective-taking does not only increase empathy for specific people, but can increase empathy toward entire stigmatized groups. For example, Batson and colleagues [3] found that participants who were asked to take the perspective of a member of a stigmatized group (e.g., people diagnosed with AIDS or convicted criminals) reported more positive attitudes not only toward a specific member of that group, but toward the stigmatized group as a whole.

The literature suggests that there are differential effects depending on the type of perspective-taking employed. For example, past studies have demonstrated that *imagine-other* perspective-taking tasks, where participants are instructed to imagine how someone else feels about a specific situation, lead to empathy and an altruistic motivation to help. On the other hand, *imagine-self* perspective-taking tasks, where participants are instructed to imagine how they would feel if they were in someone else’s situation, lead to empathy, personal distress, and an egoistic motivation to help [17–18]. Lamm, Batson, and Decety [19] have also found that there are differences in the neural mechanisms that are activated when imagine-self vs imagine-other perspective-taking tasks are used. Batson and colleagues [17] suggest that if the goal of the perspective-taking task is to maximize motivation to help, employing an imagine-self perspective-taking task may be more effective than an imagine-other approach.

Despite the general consensus that perspective-taking leads to positive outcomes [3, 13], under certain circumstances, perspective-taking can backfire and not only negate the positive outcomes outlined above, but also increase the stereotyping of others [20–22]. Perspective-taking may even cause people to blame members of a stigmatized group for their own situation even when they may not be at fault [3], and generate adverse effects in competitive settings. For example, Pierce and colleagues [13] demonstrated that taking the perspective of a competitor led to more unethical behaviors, further confirming that the positive effects of perspective-taking are contextually bound.

### Media and empathy

Apart from traditional perspective-taking, researchers have tried to promote empathy through a variety of mediated perspective-taking tasks (i.e., print media, interactive narratives, video games, and online interfaces that prompt users to take on a perspective other than their own). Mediated perspective-taking tasks provide additional information to the participant or user about a specific situation instead of relying solely on the user’s imagination. This may be particularly helpful when participants have had limited contact with or have very little or erroneous information about a social target [23]. However, it is important to note that each type of media provides information through specific channels that rely on one or a combination of the human senses. A book, for example, provides visual content and therefore relies on the visual system. A video game, however, provides visual, auditory, and sometimes haptic stimuli to the user, and therefore relies on the visual, aural, and proprioceptive systems.

Different types of media also vary in their level of immersion and interactivity. Immersion is an objective and descriptive measure of the extent to which a particular medium is able to create and sustain a “vivid illusion of reality” [24]. Interactivity refers to the extent to which users can exert influence over the content of the mediated environment in real-time [25]. The fact that different types of media such as books, TVs, computers, and VR fall under different levels of immersion and interactivity, may help explain why extant research has mixed results regarding mediated perspective-taking tasks and their effects on empathy and prosocial behaviors.

When it comes to print media, specifically the sharing of information via text, past research shows that providing new information about specific events (e.g., natural disasters) or groups of people (e.g., Native Americans) reduces social distance, increases empathy, and often results in prosocial behaviors [26–27]. Oliver and colleagues [28] highlight that the way in which information is formatted affects reader’s empathic responses, and found that information formatted as a narrative elicited more empathic responses from readers than fact-driven stories. However, providing new information does not always change people’s stereotypes of others or mitigate implicit biases [29].

Interactive interfaces such as virtual narratives (e.g., online role playing games) and video games have also been considered effective platforms to promote empathy [30–31]. For example, Hailpern and colleagues [32] created an interactive system that emulated the effects of aphasia through the real-time distortion of written text. This system gave participants a first-hand experience of what it would be like to have aphasia. Participants who interacted with the system reported feeling more empathetic and being more understanding of people with speech impediments than their counterparts who did not interact with the system at all. These interfaces are more immersive than print media because they give the user the ability to interact with digital representations of other users or algorithms (i.e., avatars or agents) and with their online environment in real-time. However, it is important to note that not all simulations of disabilities lead to positive results. For example, Brown [33] simulated schizophrenia by having participants listen to intrusive and distracting sounds for 15 minutes and found that participants’ attitudes toward schizophrenics deteriorated after the simulation. In a different study, researchers simulated blindness by covering participants’ eyes. Their results found that even though the simulation led to empathic concern, it also spread misinformation and reinforced stereotypes [34].

Higher interactivity has been linked to higher feelings of presence, or the feeling of “being there” [25], and can provide a more engaging and personalized experience [35]. Vorderer, Knobloch and Schramm [36] explored the effects of three different levels of interactivity (none, little, high) on empathy and entertainment while participants watched a movie. Results showed that, for participants with higher cognitive ability, increased interactivity led to greater empathy toward the movie’s protagonist. This study suggests that higher levels of interactivity, as a feature of immersion, can increase empathy. Using a different media platform, Behm-Morawitz, Pennel, and Speno [37] created a digital gaming app designed to reduce prejudice toward African Americans. White participants, who interacted with the game using Black avatars reported having more favorable beliefs toward African Americans and were more willing to support “pro-minority” initiatives compared to participants who used White avatars. The collective results of these studies provide evidence in support of mediated perspective-taking as an effective way to promote empathy.

### Perspective-taking tasks and VR

An IVE is a fully immersive and interactive computer-generated environment that gives the user the feeling of being somewhere other than where they are in the physical world. VR systems block out the perceptual input from the real world and replace it with perceptual input from a virtual environment that surrounds the user, is fully responsive to the user’s actions, and elicits feelings of presence. Because of these affordances, VR allows users to vividly and viscerally experience any situation as if it were happening to them from any perspective [38]. Unlike traditional media, the high level of immersion, feeling of presence, and the ability to vividly experience any situation from any perspective, may uniquely position VR as an effective perspective-taking medium.

Additionally, imagining what it would be like to be someone else is cognitively taxing [39–40]. When performing a VR perspective-taking task, however, fewer mental resources are required to create a specific environment or situation because it is all rendered digitally, and the user can solely focus on acting and reacting within the experience. This positions VR perspective-taking at an advantage in terms of accurate content, in comparison to traditional perspective-taking, since participants do not have to rely on their preexisting schemas or biases [41]. It is also methodologically advantageous since it makes it possible for all participants to undergo the exact same experience, as opposed to the lack of experimental control that occurs when participants use their imagination during traditional perspective-taking tasks [42].

Embodied cognition theory postulates that cognition is an interaction of the body and mind [43] that takes place within the context of a specific environment [44]. Past studies show that physical movement can improve a participant’s performance while completing cognitive tasks, and that the physical experience of a particular environment can have an effect on both perceptions and behaviors [45–46]. VR allows users to move and interact with their surroundings as if they were actually there through a combination of physical body movements (e.g., walking, extending their arms to reach an object, or turning their head around to examine their surroundings) and button presses using controllers. These affordances, not available in less immersive media, allow users to gather spatial information about the virtual environment using the same perceptual systems they would use to gather spatial information about the real world. Furthermore, past research has shown that the gathering of more perceptual information results in a more accurate mental representation of the physical environment [47]. Thus, the user’s ability to actively engage with and move around inside an IVE may result in improved cognition due to the additional information users are able to collect through physical movement [48], and may have a positive effect on empathy and prosocial behaviors.

In previous work, embodying the perspective of other groups (e.g., colorblind, schizophrenic, or elderly individuals) has increased helping behaviors and decreased prejudice against outgroup members [5, 49–50]. One study specifically compared the effectiveness of a traditional perspective-taking task and a VR perspective-taking task at mitigating ageism [40]. The results showed that the negative effect of threat on ageism was more effectively reduced by the VR perspective-taking task than by imagination alone. However, when the participants felt they were personally threatened by the elderly, neither VR perspective-taking nor imagination mitigated ageism.

Past studies have also shown that when light-skinned participants embody a dark-skinned avatar (i.e., a digital representation of the user) and interact in a virtual environment, implicit biases toward dark-skinned people are significantly reduced [51–52]. In a different line of studies, Ahn, Bailenson, and Park [53] compared the effects of cutting down a tree in a traditional perspective-taking task, a VR perspective-taking task, and a less immersive, mediated perspective-taking task where participants watched a video of someone cutting down a tree from the first-person perspective. Results showed that participants in the VR condition reported higher environmental behavior intentions and locus of control than their counterparts. After a week, these effects persisted for participants in the VR condition but deteriorated for the other conditions. A different study showed that embodying animals (e.g., a cow going to the slaughter house or a coral suffering the effects of ocean acidification) led to higher self-nature overlap when participants performed a VR perspective-taking task rather than just watching a video. After one week of treatment, however, the positive effect of VR mitigated [38]. To our knowledge, these are the only extant studies that have looked at the effect of VR perspective-taking past the day of treatment [38, 51–53].

Despite the encouraging results outlined above (e.g., VR perspective-taking leads to more positive outcomes than traditional perspective-taking or less immersive mediated perspective-taking tasks), these studies are limited by their small sample size (on average, less than 30 participants per condition). In fact, most psychological studies using VR systems have been conducted in laboratories where sample sizes are small and mostly composed of college students with little demographic variance [54].

In order to address the methodological issues of sample homogeneity and external validity, this investigation differs from past studies in that we gathered a large sample of data from a more diverse population by taking a mobile lab unit outside of the lab and into more naturalistic settings. For this investigation, in addition to recruiting participants in a medium-sized western university, the mobile lab unit was taken to museums, shopping centers, senior citizen centers, schools, and tech fairs with the goal of recruiting participants who represent a part of the population that has not yet been represented in psychological VR studies.

Additionally, previous studies have used self-report data to measure future intentions (e.g., 40 and 53), and while these measures often align with actual behavior, that is not always the case. For example, Rosenberg, Baughman, and Bailenson [55] measured intention to help and actual helping behaviors, and although the participants in the experimental condition exhibited more helping behaviors than those in the control condition, there was no significant difference in self-reported intention to help between the two conditions. Similarly, Bailenson and colleagues [56] found no differences in their self-report measures but detected significant differences when examining nonverbal behavioral measures. In order to address these issues and gain a more thorough understanding of the effects of different types of empathy interventions, this investigation employs both self-report and behavioral measures.

## Overview of studies

Two studies were conducted in order to compare the effects of different types of perspective-taking interventions on empathy and prosocial behaviors. Study 1 was a longitudinal investigation that compared the effects of a VR perspective-taking task against a traditional, narrative-based perspective-taking task. In Study 2, we sought a more nuanced approach to further explore the mechanisms that caused differences, or lack thereof, between the traditional and the VR perspective-taking tasks. In order to do so, we allowed for contrasts between a larger set of control conditions and developed four different empathy interventions: a fact-driven information intervention, a narrative-based perspective-taking task, and two mediated perspective-taking tasks (low vs. high immersion) in order to more accurately explore the effect of immersion and type of empathy intervention on elicited empathy and prosocial behaviors toward the homeless.

The homeless population was chosen as the social target in this work because the homeless are considered an extreme outgroup that people often struggle to empathize with [57–58]. In addition, the distinction between the in-group and outgroup is clearly defined and, unlike race or gender, it applies to a larger population because, under specific circumstances, anyone can become homeless.

## Study 1

Study 1 compared the short and long-term effects of a traditional, narrative-based perspective-taking (NPT) task and a VR perspective-taking (VRPT) task at the time of the intervention and over the course of eight weeks. As outlined above, an extensive line of research shows that perspective-taking is a powerful exercise that often results in increased empathy and prosocial behaviors toward a specific social target (e.g., stigmatized groups). However, little is known about the duration of these effects. Past research examining the longitudinal effects of perspective-taking tasks (both traditional and mediated) has only looked 1 to 2 weeks after the time of the intervention [3, 38, 51–52].

Since VR affords vivid experiences in a cognitively effortless way, a perspective-taking task through the use an IVE may be more effective at promoting empathy and prosocial behaviors than a traditional, narrative-based perspective-taking task. Additionally, participants in IVEs are surrounded by virtual stimuli that they can interact with, which may make them feel greater presence inside the virtual environment. Thus, we predict VRPT will be more effective than NPT at eliciting empathy and prosocial behaviors at the time of the intervention and over the course of eight weeks.

## Method

All procedures and materials were approved by the Ethical Committee of the Institutional Review Board at Stanford University, IRB-34677. Written consent was obtained from all participants. The individual in this manuscript has given written informed consent (as outlined in PLOS consent form) to publish these case details.

### Participants

Individuals recruited from a medium-sized western university and from around the San Francisco Bay Area formed an initial sample of 130 participants. Participants received a $100 Amazon gift card for completing the four parts of the study. All participants who agreed to participate were asked to come into the laboratory to complete the first part of the study. Of the 130 participants, 13 participants failed to complete some part of the study and were excluded from the analysis. The final sample (*N* = 117) consisted of 40 men, 75 women, and 2 participants who identified as other. The ages ranged between 15 and 57 (*M* = 22.94, *SD* = .95). Of these 117 participants, 33 (28%) were White Caucasian, 7 (6%) were Hispanic, 8 (6.8%) were Indian, 49 (41.8%) were Asian, 7 (6.8%) were African American, and 13 (11.1%) were multiracial.

### Design and procedure

In this mixed design, participants were randomly selected into one of two conditions, either the NPT (*n* = 56) or VRPT (*n* = 61) condition. All participants completed a pre-intervention questionnaire which included demographic questions, the Interpersonal Reactivity Index (IRI), and the Beliefs about Empathy scale. These scales were used in order to conduct sample checks and make sure that random assignment was successful. Participants then completed either the NPT or the VRPT task.

In both conditions, participants performed an *imagine-self* perspective-taking task. An imagine-self perspective taking task was chosen over an imagine-other task to ensure that the NPT and VRPT tasks were qualitatively the same apart from the medium being used to deliver the intervention. In the NPT condition, participants imagined what it would be like if they became homeless, and in the VRPT condition, participants experienced what it was like to become homeless inside an IVE from the first-person perspective. However, in order to control for the level of detail, accuracy of information, implicit biases against the homeless, and the scenarios that the participants would either imagine in a traditional perspective-taking task or virtually experience in the VRPT task, we standardized a narrative across the two conditions to guide participants through their respective perspective-taking tasks.

The narrative begins with the participants sitting in their apartment after losing their job and realizing rent is due. Despite selling most of their belongings, participants are not able to raise enough money to pay rent and are evicted from their apartment. Forced to live out of their car, they prepare themselves for the night by trying to find their toothbrush and other items needed to brush their teeth. Participants suddenly hear a police siren and are approached by a police officer who discovers the participants are living out of their car. Due to a city ordinance prohibiting the use of cars as homes in public spaces, the car is impounded. Participants are now traveling on a bus at night for shelter and warmth, when they are warned that there are two men onboard who may serve as a threat to them. One man may try to get unpleasantly close to the participant while the other may try to steal the participant’s backpack. In the bus, participants also interact with other non-threatening homeless people and learn about their experiences. The events in this narrative were adapted from *Hotel 22*, a documentary depicting how the homeless use public transportation for shelter at night [59], and existing interviews with homeless people in the local area. The narrative represents the lived experiences of veterans, families crippled by medical bills, victims of domestic violence, and drug addicts.

In the NPT condition, participants were given a narrative version of the storyline described above. The narrative was a written account from a first person point of view of what it would be like to become homeless (see Text A in S1 Appendix for full version). Before participants read the narrative, they were instructed to imagine the content of the narrative as if it was happening to them. The instructions and method were adapted from Batson et al. [3].

In the VRPT condition, participants experienced what it was like to become homeless inside an IVE. The narrative that the participants followed was the same narrative from the NPT condition. Participants were seated throughout the experience, but navigated the IVE by moving their head and leaning their torso to look where they wanted, and selected objects by first looking at the object they wanted to interact with and then clicking on their mouse.

**Fig 1:**
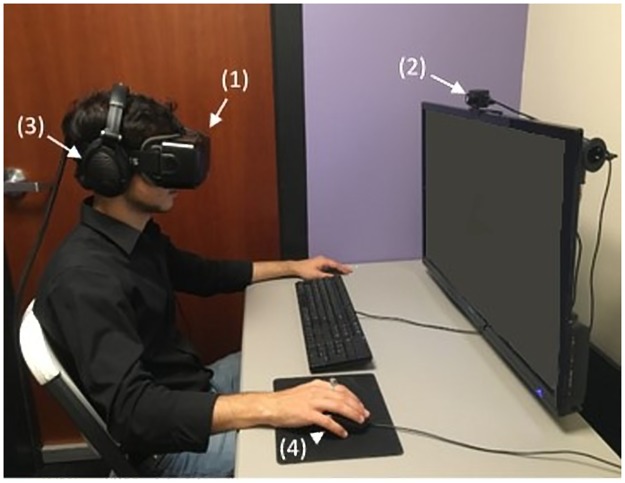
Equipment used for VRPT condition. (1) Oculus DK2 (2) Oculus DK2 infrared camera (3) headphones & (4) mouse. The individual in this manuscript has given written informed consent (as outlined in PLOS consent form) to publish these case details.

Participants in this condition wore a lightweight head-mounted display (HMD) with a resolution of 960 x 1080 pixels and a refresh rate of 75 frames per second for each eye with an average latency of 13ms. The HMD used was an Oculus Rift DK2 (Fig 1). Participants’ head translation (x, y, z position) and orientation (yaw, pitch, and roll) was tracked throughout the experience using an infrared camera (Oculus DK2 IR camera).

**Fig 2:**
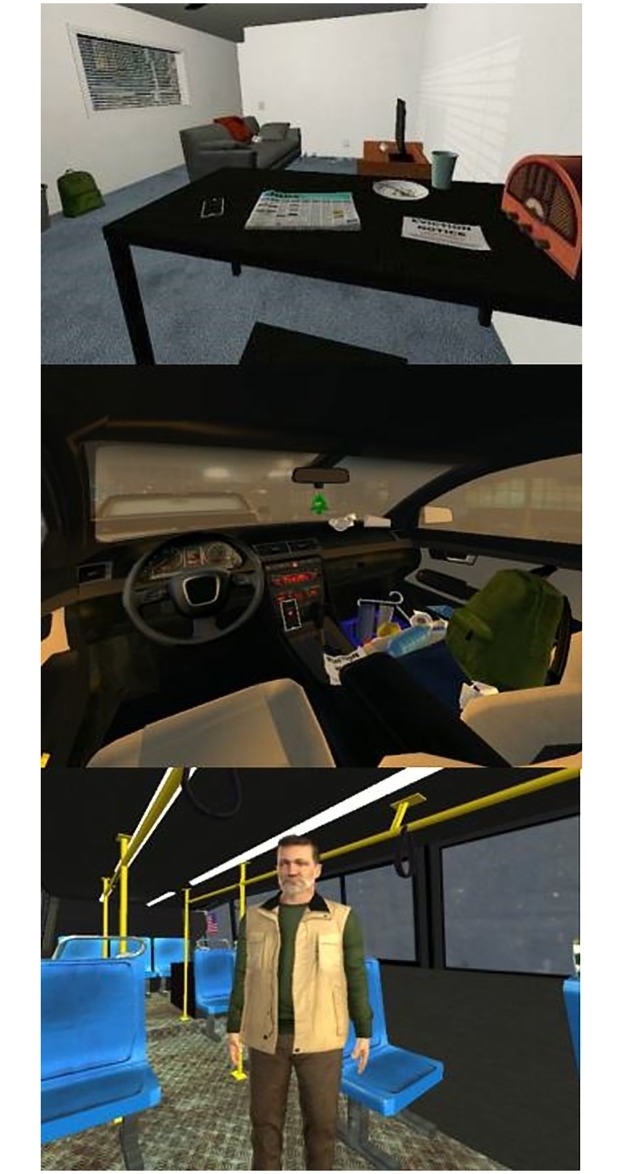
Scene progression for VR perspective-taking task. Top: Participant’s point of view in the apartment scene. Middle: Participant’s point of view in the car scene. Bottom: Participant’s point of view in the bus scene.

Unlike the NPT condition, where participants imagined what it was like to become homeless, participants in the VRPT condition experienced the narrative events described above inside the IVE. The VR experience consisted of three scenes that matched the three locations described in the narrative. This perspective-taking task differed markedly from the NPT condition in that participants in this condition were able interact with certain objects in the virtual environment. Each scene contained an interactive task where participants had the opportunity to make choices and see the consequences of those choices in real-time. For example, participants in the NPT condition were told to imagine how difficult it would be to find their toothbrush in their car, whereas participants in the VRPT condition had to actually find the toothbrush inside the car by moving their head to look around the car. None of the choices made by participants affected the length or the course of the overall VR experience. Fig 2 shows the three scenes from the participant’s point of view.

Both perspective-taking tasks, regardless of delivery method, lasted approximately 15 minutes. Upon completion of the post-intervention questionnaire, participants left the laboratory and received three follow up surveys over the next eight weeks. The follow up surveys were sent via email exactly two, four, and eight weeks after the intervention took place. Each follow-up survey was automatically sent by the Qualtrics platform as a link that would expire after 24 hours of being sent. When participants completed the surveys, researchers were able to download the data from that same platform without any identifying information.

Participants who did not meet the deadline were excluded from the analysis. At Time 0 (immediately after the intervention) 2 participants were excluded due to technical difficulties. At Time 1 (2 weeks after the intervention), 5 participants failed to complete part two within the given time frame. At Time 2 (4 weeks after the intervention) 2 participants did not complete part three. At Time 3 (eight weeks after the intervention) 4 participants did not complete the survey within the allotted time. All participants were debriefed once the study had been completed.

### Measures

#### Population variables

Interpersonal Reactivity Index. The Interpersonal Reactivity Index (IRI) is a 28-item scale with four subscales answered on a 5-point Likert scale ranging from “does not describe me well” to “describes me well”. The IRI measures individual differences in empathy by assessing the participant’s tendency to adopt the point of view of others (*M* = 3.86, *SD* = 0.57, *Cronbach’s alpha* = .77), empathic concern for others (*M* = 3.97, *SD* = 0.59, *Cronbach’s alpha* = .80), and personal distress in tense situations with others (*M* = 2.7, *SD* = 0.83, *Cronbach’s alpha* = .87) [60]. Sample items include “I sometimes find it difficult to see things from the ‘other guy’s’ point of view” and “I am often quite touched by things that I see happen”. There were two main reasons why the fantasy subscale was excluded. The first and main reason for excluding the fantasy subscale was that this was a mobile experiment. One of the limitations of having a mobile VR lab and running with volunteers (mostly composed of attendees at museums and events) is that time is an issue for most participants. Given we were already asking multiple questionnaires and the intervention lasted 15 minutes, we needed to be very selective about what questions to include. The second reason is that the fantasy subscale taps into a participant’s ability to “transpose themselves imaginatively into the feelings and actions of fictitious characters from books, movies, and plays” [60]. The fantasy subscale specifically measures the ability to embody (through imagination) a fictional character and feel what they are feeling, something that was less relevant to our intervention. We did not expect differences in any of the IRI’s subscales across conditions since they were collected before treatment.

Beliefs about Empathy Scale. The Beliefs about Empathy scale is a 12-item scale answered on a 7-point Likert scale (1 = strongly disagree, 7 = strongly agree) that measures the extent to which people believe that empathy is something that can be controlled. Sample items include “people can always change how much empathy they generally feel for others” and “when a person feels empathy for someone, they can’t stop feeling empathy, even if they want to stop.” The scale contains two different subscales, controllability (*M* = 4.31, *SD* = 1.06, *Cronbach’s alpha* = .82) and implicit theories (*M* = 4.51, *SD* = 1.33, *Cronbach’s alpha* = .92). The overall reliability of this scale was good, *Cronbach’s alpha* = .87. This scale is used as a sample check to make sure that there are no significant differences in beliefs about empathy across conditions, as previous research has demonstrated that participants who believe they can control their empathic responses actually exert more empathic effort than those who believe they have no control [61].

#### Outcome variables

Manipulation Check. In order to assess if participants in the VRPT condition felt more spatially present than participants in the NPT condition, participant’s open-ended responses to the question “What are your thoughts about your experience so far?” were coded. Two coders, blind to condition, coded the participants’ responses independently. The coding scheme employed was deductive and adapted from the presence questionnaires used in Nowak and Biocca [62] and Bailenson and Yee [63]. Participants received a 1, 0, or -1 depending on their responses.

Participants received a “1” if they explicitly mentioned that the experience or narrative felt real, was realistic, or immersive, if they felt immersed, “there”, inside the environment, or if they were physically or spatially affected by what they experienced in VR or what they imagined. Participants received a “0” if their response was not related to presence or immersion, and a “-1” if they explicitly mentioned that the experience or narrative was not real or realistic, that they did not feel “there”, immersed, or physically affected by what they experienced in VR or what they imagined (*M* = 0.15, *SD* = 0.47).

Inclusion of the Other in the Self. The Inclusion of the Other in the Self (IOS) scale is a single-item, pictorial measure of closeness and connectedness. In the IOS scale, participants select the picture that best represents their relationship with the average homeless person [64]. Pictures are Venn-like diagrams of two circles overlapping, with each circle representing a homeless person and the self, respectively. The pictures are coded from 1 to 7 with the larger numbers indicating a closer relationship with the homeless (i.e., a larger degree of overlap between the two circles) (*M* = 3.09, *SD* = 1.92).

Empathy. Using a 7-point Likert scale (1 = Not at All, 7 = Extremely), participants were asked to rate the extent to which they felt *softhearted*, *touched*, *sympathetic*, or *compassionate* throughout the intervention. The results of these four questions were used to create an index of empathic concern. This measure was adapted from Batson, Early and Salvarani [17]. The overall reliability of the index was good, *Cronbach’s alpha* = .88 (*M* = 5.05, *SD* = 1.21).

Personal Distress. Using a 7-point Likert scale (1 = Not at All, 7 = Extremely), participants were asked to rate the extent to which they felt *uneasy*, *troubled*, *distressed*, or *disturbed* throughout the intervention. The results of these four questions were used to create an index of personal distress. This measure was also adapted from Batson, Early and Salvarani [17]. The overall reliability of the index was good, *Cronbach’s alpha* = .87 (*M* = 4.58, *SD* = 1.33).

Dehumanization Scale. The Ascent of Man measure of Dehumanization is a single-item measure of blatant dehumanization [65]. This measure was chosen to examine whether or not traditional or VR perspective-taking tasks could prevent dehumanization toward the homeless since past research has demonstrated that people are willing to overtly describe outgroup members as less evolved than members of their own group [57–58, 65]. Participants are asked to rate how evolved the average member of the homeless population is by looking at the “Ascent of Man” evolution picture that depicts a primate evolving into a human (0 = not evolved/primate, 100 = fully evolved/human) (*M* = 89.06, *SD* = 16.09).

Social Presence Scale. The 6-item social presence scale assesses how present participants felt with virtual humans inside the IVE in the VRPT condition and was adapted from Nowak and Biocca [62]. Participants were asked to rate how strongly they felt the virtual humans were with them inside the IVE using a 5-point Likert scale (1 = not at all, 5 = very strongly). The reliability of the scale was good, *Cronbach’s alpha* = .92 (*M* = 1.49, *SD* = 1.55). Sample items include “How strongly did you sense that the virtual humans were aware of your presence” and “How strongly did you sense that the virtual humans were present?”

Attitudes toward the Homeless. The 7-item attitudes scale was answered on a 9-point Likert scale (1 = strongly disagree, 7 = strongly agree). This scale adapted from Batson et al. [3] to gauge attitudes toward the homeless (*M* = 6.48, *SD* = 1.18, *Cronbach’s alpha* = .83). Sample items include “Most homeless people could have avoided becoming homeless” and “Our society does not do enough to help homeless people.” Two out of the seven items from the attitudes toward the homeless scale were reverse coded. Higher scores indicate more positive attitudes toward the homeless.

#### Behavioral measures

Agreement with Proposition A. Proposition A, a real proposition at the time the study was conducted, supported increasing affordable housing for vulnerable populations in the San Francisco Bay Area (Text B in S1 Appendix). After participants read Proposition A, they were asked the extent to which they agreed with the proposition using a 5-point Likert scale (*M* = 4.07, *SD* = .76) ranging from “not at all” to “completely”. This measure was implemented at Time 0 (immediately after the intervention).

Signing Petition Supporting Proposition A. Participants were also asked whether or not they were willing to sign a petition supporting Proposition A. At this point, participants were reminded that the passing of the proposition would mean an increase in their taxes. Instead of simply selecting *yes* or *no*, participants either signed the petition or left the petition page blank (71.8% of all participants signed the petition). The researcher was not in the room while the participant read or responded to the petition, so as not to influence the participant’s behaviors. This measure was also implemented at Time 0 and participants were debriefed that the petition was not a real petition upon completion of the study eight weeks later.

Donation Question. After either empathy intervention, participants were asked if they wanted to donate part of their compensation to a homeless shelter using an 11-point Likert scale ranging from “$0” to “$10” (*M* = 5.73, *SD* = 3.78). Participants were compensated in full regardless of whether they chose to donate any money or not. However, participants were not aware they were going to receive full compensation until they were debriefed after the study had been completed. Again, the researcher was not in the room with the participant while they chose whether or not to donate money. This measure was also implemented immediately after the intervention.

Letter Writing. Participants were asked to write a letter to their elected officials regarding the issue of homelessness at Time 1 (2 weeks after the intervention), and a letter to a friend describing how they felt and what they had learned about the issue of homelessness at Time 3 (eight weeks after the intervention). For instructions see Text C in S1 Appendix. These behavioral measures were added in order to 1) examine the extent of each participant’s civic engagement regarding the issue of homelessness, and 2) conduct linguistic analysis to further understand participants’ emotional states through their written language. Past research demonstrated that analyzing the way that people write (function words) rather than what they write about (content words), is a reliable measure of different psychological and emotional states [66]. We focused on analyzing 7 categories: word count, positive emotion, negative emotion, social, anxiety, I, and we. These categories were specifically chosen to quantify affect, and examine the extent to which participants included or excluded themselves as part of the solution when writing about the issue of homelessness [67]. All linguistic analyses were performed using Linguistic Inquiry and Word Count (LIWC) software.

Agreement with Measure B. Similar to Proposition A, Measure B also advocated for increasing affordable housing for vulnerable populations (Text D in S1 Appendix). This measure provided different, yet related, information about what can be done to help the homeless in order to assess support for helpful initiatives over time. This behavioral measure was implemented at Time 2 (4 weeks after the intervention). Participants were asked to read Measure B and report the extent to which they agreed with it using a 5-point Likert scale (1 = not at all, 5 = completely) (*M* = 4.19, *SD* = .92).

## Results

### Population variables

A one-way analysis of variance (ANOVA) showed that there was no significant difference in participants’ trait-levels of empathy on any subscale of the IRI scale across the two conditions (Perspective Taking: *t*(112) = 0.30, *p* = .762; 95% Confidence Interval (CI) [-.18, .24], Empathic Concern: *t*(108) = -0.39, *p* = .697; 95 CI [-.26, .18], and Personal Distress: *t*(114) = 0.39, *p* = .695; 95 CI [-.24, .36]). There were also no significant differences between the two conditions regarding beliefs about empathy (Controllability: *t*(113) = 0.11, *p* = .912; 95 CI [-.37, .41], Implicit Theories: *t*(114) = -1.25, *p* = .214; 95 CI [-.79, .18]). These results show that there was a balance across conditions in terms of the way that people think about empathy and in the way that they believe they are able to control their empathic responses, showing that random assignment was successful across conditions.

### Outcome variables

#### Manipulation check

Cohen’s Kappa was calculated in order to assess inter-coder reliability and agreement between the two coders [68]. There was substantial agreement between the two coders (κ = .89, *p* < .001, 95% CI [.81, .99]. Data from the two coders were averaged together. A Chi-Squared test was used in order to test whether or not there was a significant difference in the proportion of participants who reported feeling present (values equal to 1) and the participants who did not (values smaller than 1) across conditions. In the NPT condition 5.4% of participants received a score of 1 and in the VRPT condition 34.4% participants received a score of 1. Results showed that significantly more participants in the VRPT condition reported feeling spatially present than participants in the NPT condition (*X*^*2*^(1) = 15.06, *p* < .001; 95 CI [14.98, 42.10]).

#### Continuous variables

The means and standard deviations for the outcome variables (i.e., IOS, Dehumanization, Empathy, Personal Distress, Social Presence, support for Proposition A, support for Measure B, and amount donated) by condition and time are summarized in Table 1.

**Table 1:** Means and standard deviations for all outcome variables by condition and time.

	**NPT Condition**							
Measures	Time 0		Time 1		Time 2		Time 3	
	*Mean*	*SD*	*Mean*	*SD*	*Mean*	*SD*	*Mean*	*SD*
IOS	2.84	1.99	2.42	1.44	2.65	1.63	2.68	1.73
Dehumanization	89.3	15.3	87.86	18.21	88.85	12.7	87.21	19
Empathy	4.8	1.2	4.57	1.05	4.53	1.14	4.43	1.12
Personal Distress	4.4	1.26	4.38	1.1	4.15	1.26	4.21	1.31
Attitudes	6.39	1.14	5.98	1.27	6	1.24	6.07	1.26
Support Proposition A	4.09	0.83	-	-	-	-	-	-
Amount Donated	5.44	3.72	-	-	-	-	-	-
Support Measure B	-	-	-	-	4.14	0.89	-	-
Petition Signing Proportion	35 out of 57							
	**VRPT Condition**							
Measures	Time 0		Time 1		Time 2		Time 3	
	*Mean*	*SD*	*Mean*	*SD*	*Mean*	*SD*	*Mean*	*SD*
IOS	3.33	1.87	2.97	1.68	3.08	1.83	3.1	1.8
Dehumanization	88.8	16.96	91.28	12.86	91.3	12.71	91.4	15.08
Empathy	5.29	1.18	4.9	1.07	4.87	1.2	4.99	1.22
Personal Distress	4.75	1.38	4.63	1.15	4.6	1.28	4.77	1.25
Presence	2.9	0.74	-	-	-	-	-	-
Attitudes	6.56	1.22	6.54	1.21	6.4	1.22	6.35	1.4
Support Proposition A	4.07	0.69	-	-	-	-	-	-
Amount Donated	6.02	3.86	-	-	-	-	-	-
Support Measure B	**-**	-	-	-	4.23	0.96	-	-
Petition Signing proportion	49 out of 60							

*SD* = Standard deviation.

All continuous outcome variables were analyzed using a linear growth curve model with fixed effect of condition (NPT vs VRPT) on the intercept (results right after the intervention) and linear terms (trend over the course of eight weeks). Random effect of individuals on the intercept and the slopes were included in the models as well. Growth curve modeling analysis was chosen since it accounts for inter-participant variability (between) and intra-participant (within) patterns of change over time [69–70]. All analyses were carried out in R version 3.0.2 using the nlme package.

**Fig 3:**
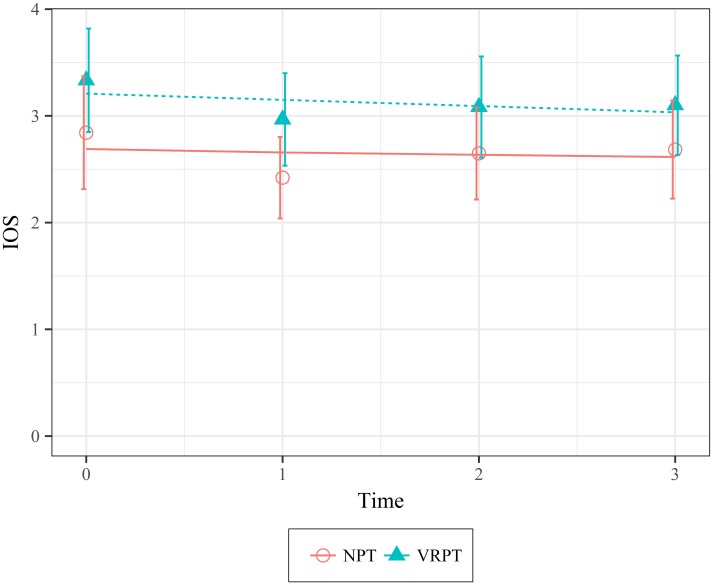
Mean values of self-other overlap with the homeless as a function of condition over time. Higher scores represent higher overlap between the self and the homeless. Error bars represent 95% Confidence Intervals.

IOS. There were no statistically significant differences in self-other overlap between the conditions at the time of the intervention (Time 0) (*X*^*2*^(1) = 2.22, *p* = .136; 95 CI [-0.15, 1.08]). There was also no effect of condition on how connected participants felt toward the homeless in the eight weeks following the intervention (*X*^*2*^(1) = .1024, *p* = .749; 95 CI [-0.23, .17]). Fig 3 shows the data and model fit. The general trend for both conditions is that participants felt moderately connected to the homeless immediately after the intervention, but experienced a slight decline of .03 points (*SE* = 0.1) in perceived connectedness at the time of each follow up.

**Fig 4:**
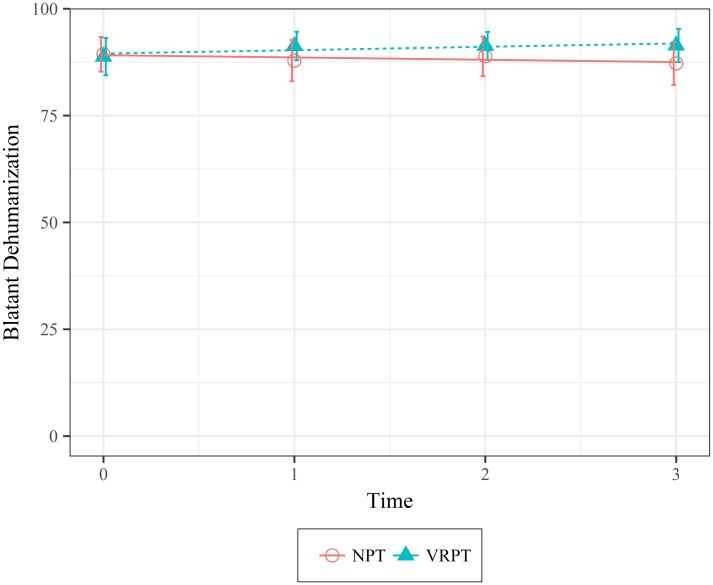
Mean values of blatant dehumanization of the homeless as a function of condition over time. Error bars represent 95% Confidence Intervals.

Dehumanization. There was no significant effect of condition on blatant dehumanization at the time of the intervention (*X*^*2*^(1) = .32, *p* = .572; 95 CI [-5.22, 5.68]). However, there was a significant effect of condition indicating that, over time, participants in the VRPT condition thought of the homeless as more evolved than the participants in the NPT condition who thought of the homeless as less evolved over time (*X*^*2*^(1) = 3.8, *p* = .051; 95 CI [0, 2.66]). The difference was approximately 1.33 (*SE* = .68) points biweekly showing that although participants in both conditions thought of the homeless similarly at the time of the intervention, participants in the VRPT condition thought significantly higher of the homeless over time than those in the NPT condition. Fig 4 shows the data and model fit.

**Fig 5:**
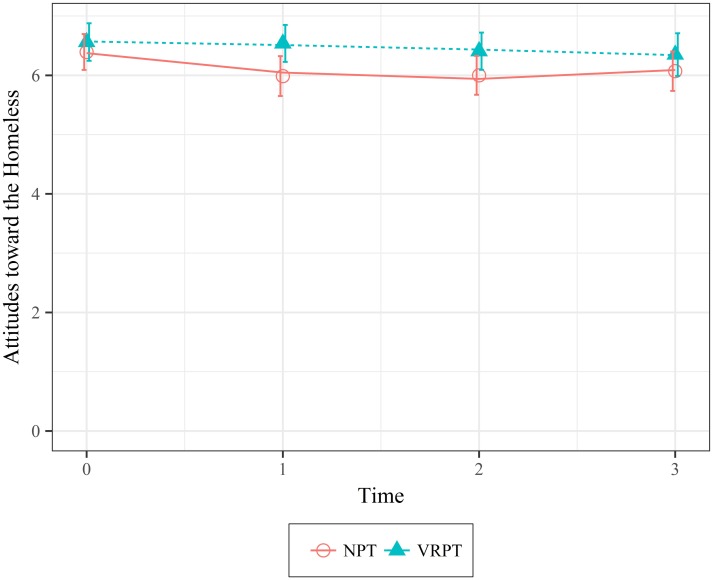
Mean values of attitudes toward the homeless as a function of condition over time. Error bars represent 95% Confidence Intervals.

Attitudes toward the Homeless. Visual inspection of the residuals of the model testing the effect of condition and time on attitudes toward the homeless revealed a curvilinear relationship between these variables. The quadratic model fit the data significantly better than the linear model (*X*^*2*^(2) = 13.37, *p* = .001; 95 CI [-5.02, -1.0]). Since two models were compared for this dependent variable, a Bonferroni correction (*p* = *α*/2) was implemented and results reflect the adjusted p-values. There was no significant difference in attitudes toward the homeless between the two conditions at Time 0 (*X*^*2*^(1) = 2.37, *p* = 0.124; 95 CI [-0.07, .75]). However, there was a significant effect of condition (*X*^*2*^(1) = 8.19, *p* = 0.033; 95 CI [-5.02, -0.9]) over the course of the eight weeks. Even though attitudes toward the homeless deteriorated over time, the attitudes deteriorated at a significantly slower rate and were consistently more favorable for participants in the VRPT condition than the participants in the NPT condition. Fig 5 shows the data and model fit.

**Fig 6:**
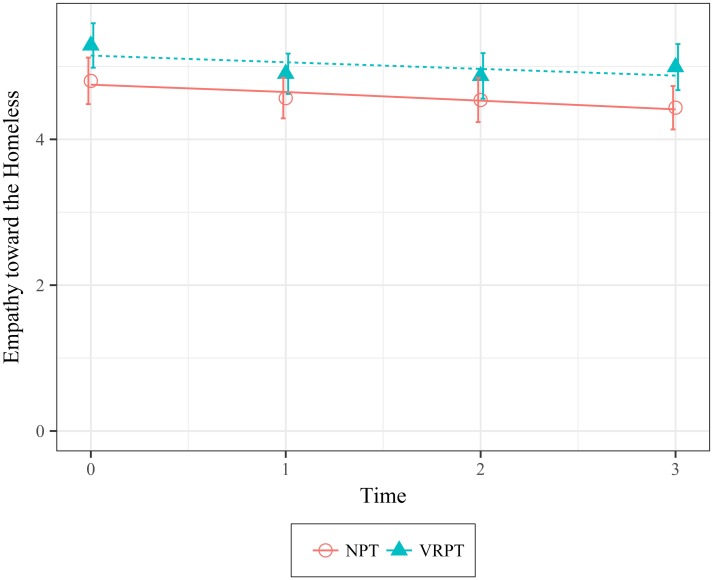
Mean values of empathy as a function of condition over time. Error bars represent 95% Confidence Intervals.

Empathy. Participants in the VRPT condition reported feeling significantly more empathetic than participants in the NPT condition immediately following the intervention (*X*^*2*^(1) = 4.53, *p* = .033; 95 CI [.04, .77]). However, there was no significant difference between the two conditions in self-reported empathy two, four, or eight weeks after the intervention (*X*^*2*^(2) = 3.08, *p* = .214; 95 CI [-.31, 4.16]). Fig 6 shows the data and model fit.

**Fig 7:**
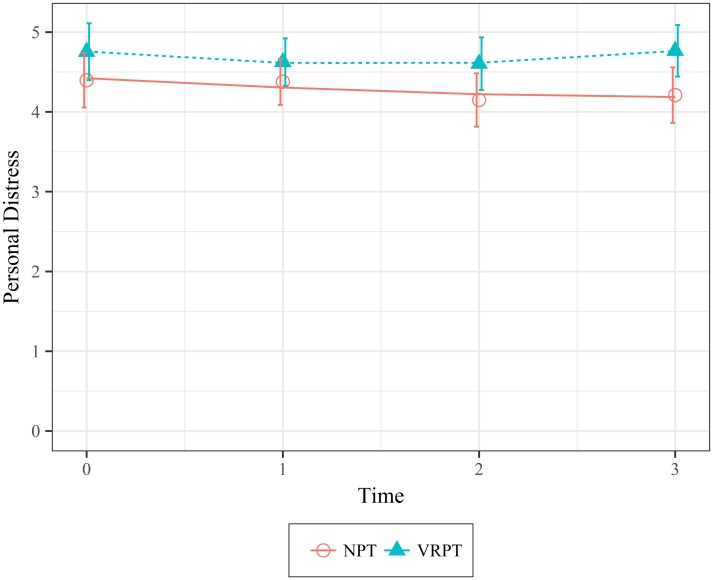
Mean values of personal distress as a function of condition over time. Error bars represent 95% Confidence Intervals.

Personal Distress. Participants in the VRPT condition reported feeling significantly more distressed and troubled after the intervention than participants in the NPT condition (*X*^*2*^(1) = 4.48, *p* = .034; 95 CI [.04, .76]). However, there were no significant differences between the two conditions over the course of eight weeks (*X*^*2*^(2) = 0.84, *p* = .359; 95 CI [-1.78, 3.88]). These results indicate that significant differences in personal distress only occurred immediately after the intervention. Fig 7 shows the data and model fit.

Correlations among Dependent Variables. All simple Pearson correlations between dependent variables are included in Table 2. A Holm-Bonferroni correction was implemented to account for multiple comparisons. Results show the adjusted p-values.

**Table 2:** Simple correlations among dependent variables.

**Time 0—Immediately After the Intervention**							
	Dehumanization	Attitudes	Empathy	Personal Distress	Social Presence	Support Prop A	Donation
IOS	0.10	0.18	0.08	-0.01	0.15	0.04	-0.01
Dehumanization		0.21	-0.07	-0.09	-0.03	-0.02	0.15
Attitudes			**0.38** ^c^	0.22	0.14	-0.04	0.08
State Empathy				**0.30** ^a^	0.25	-0.09	0.06
Personal Distress					0.16	-0.06	-0.05
Social Presence						-0.05	-0.12
Support Prop A							0.16
Donation							
**Time 1—Two Weeks After the Intervention**							
	Dehumanization	Attitudes	Empathy	Personal Distress			
IOS	0.13	**0.25** ^a^	0.07	0.04			
Dehumanization		**0.32** ^b^	0.11	0.13			
Attitudes			**0.61** ^c^	**0.27** ^a^			
State Empathy				**0.41** ^c^			
Personal Distress							
**Time 2—Four Weeks After the Intervention**							
	Dehumanization	Attitudes	Empathy	Personal Distress	Measure B		
IOS	0.19	**0.28** ^a^	0.24	0.18	0.16		
Dehumanization		**0.32** ^b^	0.25	0.09	0.12		
Attitudes			**0.68** ^c^	**0.37** ^c^	**0.40** ^c^		
State Empathy				**0.53** ^c^	0.24		
Personal Distress					0.14		
Measure B							
**Time 3—Eight Weeks After the Intervention**							
	Dehumanization	Attitudes	Empathy	Personal Distress			
IOS	**0.26** ^a^	**0.30** ^b^	0.22	0.16			
Dehumanization		**0.31** ^b^	0.12	-0.01			
Attitudes			**0.55** ^c^	**0.28** ^b^			
State Empathy				**0.46** ^c^			
Personal Distress							

^a^ positive at p < .05.

^b^ positive at p < .01.

^c^ positive at p < .001.

The correlation analysis showed that IOS scores were not significantly correlated with attitudes toward the homeless immediately after the intervention. However, IOS scores and attitudes became significantly and positively correlated over the course of eight weeks (Time 0: r = 0.18, *p* = 1.00; Time 1: r = 0.25, *p* = .039; Time 2: r = 0.28, *p* = .024; Time 3: r = 0.30, *p* = .008) such that participants who reported feeling more connected to the homeless also reported more positive attitudes toward them. Dehumanization scores were also not correlated with attitudes toward the homeless immediately after the perspective-taking tasks but became significantly and positively correlated over the course of eight weeks (Time 0: r = 0.21, *p* = .006; Time 1: r = 0.32, *p* = .003; Time 2: r = 0.32, *p* = .004; Time 3: r = 0.31, *p* = .006). The analysis also revealed that attitudes toward the homeless scores were significantly and positively correlated with empathy (Time 0: r = 0.38, *p* < .001; Time 1: r = 0.61, *p* < .001; Time 2: r = 0.68, *p* < .001; Time 3: r = 0.55, *p* < .001) such that participants who reported more empathy also reported more positive attitudes toward the homeless.

Moreover, attitudes toward the homeless were not correlated with personal distress immediately after the intervention. However, the correlation between attitudes and personal distress became significant over the course of eight weeks (Time 0: r = 0.22, *p* = .489; Time 1: r = 0.27, *p* = .026; Time 2: r = 0.37, *p* < .001; Time 3: r = 0.28, *p* = .013). The analysis also revealed that self-reported empathy was significantly and positively correlated with personal distress (Time 0: r = 0.30, *p* = .032; Time 1: r = 0.41, *p* < .001; Time 2: r = 0.53, *p* < .001; Time 3: r = 0.46, *p* < .001).

Furthermore, four weeks after the intervention, the correlation analysis revealed that attitudes toward the homeless were significantly and positively correlated with support for Measure B (r = 0.40, *p* < .001). In other words, participants who reported more positive attitudes toward the homeless also reported more support for affordable housing four weeks after the intervention. It is important to note that attitudes toward the homeless were not correlated with support for affordable housing at the time intervention (r = -0.04, *p* > 1.00). These results suggest that over time, the positive correlation between attitudes and support for helpful initiatives strengthens.

#### Behavioral measures

Behavioral Measures at time 0. There was no significant difference between conditions regarding self-reported support for Proposition A (*t* (108) = .15, *p* = .882; 95 CI [-.03, .3]). However, significantly more participants in the VRPT condition signed the petition in support of Proposition A (*z* (115) = 2.39, *p* = .023; 95 CI [1.12, 7.21]) in comparison to the NPT condition. Even though participants in both conditions claimed to support Proposition A to the same extent, a significantly higher proportion of participants who performed the VRPT task physically signed the petition in support of affordable housing. For the donation question, there was no significant effect of condition on amount donated to a homeless shelter (*t* (114) = -.82, *p* = .411; 95 CI [-1.97, .81]). On average, participants from both conditions donated about 5 dollars to a homeless shelter.

Behavioral Measures at Time 1. Two weeks after the intervention, participants were asked to write a letter to their elected official on the issue of homelessness. After analyzing the text of the letters, there were no significant differences in word count (*t* (108) = 0.64, *p* = 0.521; 95 CI [-15.58, 30.54]), positive or negative emotion (*t*(100) = 0.05, *p* = 0.960, 95 CI [-1.01, 1.06]; *t* (110) = -0.59, *p* = 0.557, 95 CI [-.87, .47]), or the ‘I’ dictionary (*t*(104) = 1.11, *p* = 0.269; 95 CI [-.38, 1.35]). However, there were marginally significant differences in the anxiety (*t*(80) = -1.78, *p* = 0.078; 95 CI [-.42, .02]) and social (*t*(107) = -1.85, *p* = 0.067; 95 CI [-3.94, .14]) dictionaries, showing that participants in the VRPT condition were more anxious while writing the letter, and used more social words than participants in the NPT condition. There was also a significant difference in the ‘we’ dictionary, indicating that participants in the VRPT condition wrote about the issue of homelessness and its possible solutions using more “we”, “our”, “us” pronouns than the participants in the NPT condition (*t*(102) = -2.01, *p* = 0.046; 95 CI [-1.53, -.01]). Means and standard deviations used for all the dictionaries by condition can be found in Table 3.

**Table 3:** Means and standard deviations for all LIWC dictionaries used.

	Time 1				Time 3			
	NPT		VRPT		NPT		VRPT	
Dictionaries	*Mean*	*SD*	*Mean*	*SD*	*Mean*	*SD*	*Mean*	*SD*
Word Count	111.98	65.1	104.5	58.16	98.32	81.4	122.65	110.49
Positive Emotion	3.87	3.12	3.84	2.37	3.46	3.39	2.96	1.91
Negative Emotion	1.95	1.67	2.15	1.92	2.11	2.18	2.41	2.21
Anxiety	0.15	0.35	0.35	0.77	0.25	0.61	0.32	0.64
Social	12.18	5.82	14.08	5.07	12.28	6.88	11.92	4.22
We	1.37	1.65	2.14	2.37	1.23	2.17	1.71	1.72
I	2.34	2.54	2.08	1.85	2.45	2.32	3.25	2.86

*SD* = Standard Deviation.

Behavioral Measures at Time 2. Four weeks after the intervention, at Time 2, participants were asked to what extent they agreed with Measure B, a measure proposed in the San Francisco Bay Area supporting affordable housing. Participants in the VRPT condition reported more support for Measure B than participants in the NPT condition (*t*(105) = -1.74, *p* = .08; 95 CI [-.63, .04]). However, these results were only marginally significant.

Behavioral Measures at Time 3. Eight weeks after the intervention, participants were asked to write a letter to a friend telling them what they had learned and thought about the issue of homelessness. After analyzing the text of the letters, there were no significant differences in any of the 7 LIWC dictionaries (word count: *t*(108) = -1.37, *p* = 0.176, 95 CI [-59.78, 11.11]; positive emotion: *t*(87) = 0.96, *p* = 0.340, 95 CI [-0.53, 1.51]; negative emotion: *t*(114) = -0.75, *p* = 0.454, 95 CI [-1.11, 0.5]; we: *t*(107) = -1.33, *p* = 0.189, 95 CI [-1.2, 0.24]; I: *t*(112) = -1.67, *p* = 0.097, 95 CI [-1.75, 0.15]; anxiety: *t*(115) = -0.67, *p* = 0.506, 95 CI [-0.31, 0.15]; social: *t*(92) = 0.34, *p* = 0.735, 95 CI [-1.75, 2.47]).

## Discussion

Both types of perspective-taking tasks led to similar results when it came to self-other overlap immediately after the intervention and over the course of eight weeks. Participants in the VRPT condition reported significantly more empathy and more personal distress immediately after the intervention. However, over time, participants in both the VRPT and NPT conditions reported similar rates of empathy and personal distress. The results for attitudes toward the homeless and the dehumanization scale show the opposite pattern. Even though both conditions reported similar rates of dehumanization and attitudes toward the homeless at the time of the intervention, participants in the NPT condition thought of the homeless as less evolved over time, and the attitudes they had for the homeless deteriorated in the eight weeks that followed the intervention. In contrast, the VRPT condition, which allowed participants to interact with the virtual environment in real-time, led to more positive, longer-lasting attitudes toward the homeless up to two months after the intervention.

Past research has demonstrated that after a perspective-taking task, participants tend to feel empathetic toward a specific target immediately after the task, but over time, the empathic feelings wane while attitudes toward that target improve [71]. In a different study, Batson et al [3] found that participants who performed a perspective-taking task felt more empathy for convicted murderers than participants who were asked to remain objective. There were no differences in attitudes toward the convicts between the two conditions immediately after the task, but there were significant differences in attitudes 1–2 weeks after the intervention, indicating that feeling empathetic toward a member of a stigmatized group may lead to better attitudes over time rather than immediately after the perspective-taking task. Batson suggests that attitudes can “outlive the empathic emotion itself” ([3] p. 116). Our results replicate these findings since participants in the VRPT condition reported more empathy but similar attitudes when compared to the NPT participants immediately after the intervention, but significantly better attitudes toward the homeless over time. Additionally, improved attitudes lasted longer than the empathic feelings themselves.

It is also important to note that while there were no significant differences when it came to self-reported support for Proposition A between the two conditions, significantly more participants in the VRPT condition signed the petition in support of affordable housing even when it meant an increase in their taxes.

Overall, these results show that immediately after the intervention, VRPT led to more self-reported empathy and personal distress. Over time, VRPT did not lead to more self-other overlap, self-reported empathy, personal distress, or donations to a homeless shelter than more traditional perspective-taking tasks. However, VRPT did result in more positive, longer-lasting attitudes toward the homeless and significantly more signatures supporting helpful initiatives than the NPT condition.

## Study 2

The results of Study 1 showed that, over time, participants in both the NPT and VRPT condition reported feeling empathetic and connected to the homeless at similar rates. However, VRPT led to more signatures supporting affordable housing and more positive, longer-lasting attitudes. These results show that perspective-taking aided by immersive VR media can effectively improve attitudes toward the homeless. However, it is possible that less immersive media, such as desktop computers and laptops, produce similar effects without the need to fully surround participants with stimuli. Study 2 expands on Study 1 by comparing the effect of four different types of empathy interventions: a fact-driven information intervention (Information), a traditional, narrative-based perspective-taking task (NPT), a VR perspective-taking task (VRPT), and a less immersive mediated perspective-taking task using a desktop computer (Desktop) in order to more accurately assess the effect of perspective-taking and examine the role immersion plays when attempting to promote empathy and prosocial behaviors.

Since past research demonstrates that perspective-taking leads to increased empathy and helping behaviors, we predict that any type of perspective-taking would be more effective at eliciting empathy and prosocial behaviors than receiving information (i.e., the three perspective-taking conditions vs. Information). Given the results obtained in Study 1, we also predict that mediated perspective-taking would be more effective than NPT (i.e. Desktop and VRPT vs NPT), and that the most immersive perspective-taking task would be more effective than the less immersive perspective-taking tasks (i.e. VRPT vs Desktop).

## Method

### Participants

A total of 452 participants were recruited to participate in this study. Thirteen participants were excluded from the analysis because they did not complete the study in its entirety. Of the remaining 439 participants (189 men, 250 women), 190 participants were students recruited from a medium-sized western university and 249 were recruited at mobile sites such as schools, museums, and senior citizen centers in the San Francisco Bay Area. Participants provided informed consent and were compensated with either course credit or a $10 Amazon gift card for participating. The mean age of the participants was 29.2 (*SD* = 14.8) and ranged between 15 and 88 years old. Of these 439 participants, 22 (5%) were African American, 9 (2%) were Middle Eastern, 27 (6%) were Indian, 5 (1%) were Native American, 86 (20%) were Asian, 32 (7%) were Hispanic, 215 (48%) were White Caucasian, 40 (9%) were multiracial, and the rest (1%) declined to answer. Participants recruited at mobile sites participated in the study inside a 3.96m x 2.13m x 2m tent where they were given complete privacy in public areas. Apart from the physical setting, all of the participants used the same equipment and followed the same procedures. There was no overlap between participants from Study 1 and Study 2.

### Design and procedure

In this between-subjects design, participants signed a consent form and were randomly selected into one of four conditions: 1) Information, 2) NPT 3) Desktop, or 4) VRPT. After random assignment, all participants completed a pre-intervention questionnaire which included demographic questions, the Interpersonal Reactivity Index (IRI), and the Beliefs about Empathy scale. These scales were used in order to conduct sample checks and make sure that random assignment was successful. Upon completing this questionnaire, participants in each condition received a different empathy intervention.

In the Information condition (*n* = 107), participants were asked to read a packet of information and statistics about the homeless population in the Bay Area in 2015 [72]. The packet was a combination of written information, graphs, tables, maps, and pie charts. The packet provided information on the main causes for homelessness, obstacles faced by the homeless when trying to get a job, as well as the percentage of the population that were children, sick, or had a history of foster care. To read all of the information provided in this packet please see Text E in S1 Appendix. After reading the packet, participants completed a post-intervention questionnaire consisting of self-report and behavioral measures. This fact-driven intervention was chosen in order to examine the effectiveness of perspective-taking (mediated or not) against a rigorous, real-world intervention that does not utilize perspective-taking at all and was specifically designed to increase awareness about the homeless.

The NPT (*n* = 104) and the VRPT (*n* = 115) conditions in Study 2 were the same as the conditions described Study 1.

**Fig 8:**
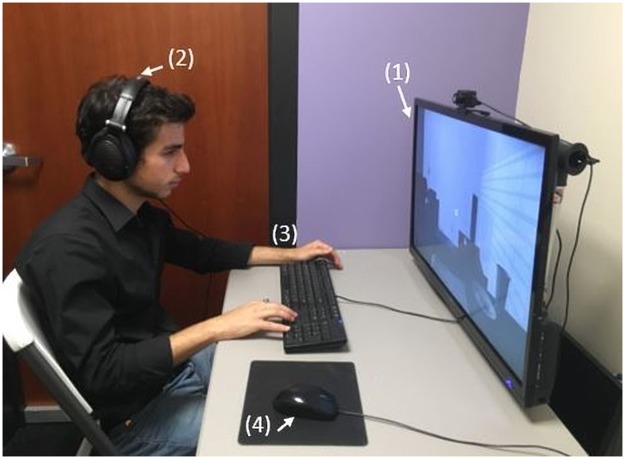
Equipment used for desktop condition. (1) TV screen (2) headphones (3) keyboard & (4) mouse. The individual in this manuscript has given written informed consent (as outlined in PLOS consent form) to publish these case details.

In the ‘Desktop’ condition (*n* = 113), participants sat in front of a computer with a flat TV screen (39”) and experienced what it was like to become homeless through a 2D interactive narrative (Fig 8). The narrative that the participants followed was the same interactive narrative from the VRPT condition, except participants were only able to view the environment on the screen, navigate the online environment using the arrow keys on the computer keyboard, and select objects with their mouse. The functionality of this interactive narrative resembled that of an online game. Participants in this condition were given the same interactive tasks as the VRPT condition, and were able to make their own choices, navigate the virtual environment, and receive immediate feedback. Fig 2 also shows the three scenes from the participant’s point of view.

All of the perspective-taking tasks, regardless of type, lasted approximately 15 minutes. All participants completed the same post-intervention questionnaire immediately after the intervention. All participants were debriefed once the study had been completed.

### Measures

The same manipulation check employed in Study 1 was used in Study 2 to assess how spatially present participants felt across the three perspective-taking conditions. The self-report measures used in Study 2 (described in Study 1) were IRI, Beliefs about Empathy, IOS, Dehumanization, Empathy, Personal Distress, and Social Presence. The behavioral measures used were self-reported support for Proposition A, whether or not participants signed a petition supporting Proposition A, and the homeless shelter donation question. The overall means and standard deviations, as well as the reliability of the scales (Cronbach’s alpha) used in Study 2 can be found on Table 4.

**Table 4:** Overall means, standard deviations, and reliability of scales used in Study 2.

Measures	*Mean*	*SD*	*α*
IRI: EC	3.92	0.59	0.76
IRI: PT	3.70	0.59	0.74
IRI: PD	2.64	0.72	0.81
Beliefs about Empathy: C	4.15	1.09	0.81
Beliefs about Empathy: IT	4.28	1.20	0.89
IOS	2.86	1.75	-
Dehumanization	89.95	16.09	-
Empathy	5.09	1.22	0.88
Personal Distress	4.15	1.43	0.85
Presence	2.88	0.82	0.80

*SD* = Standard deviation.

*α* = Cronbach’s alpha.

## Results

### Population variables

Replicating Study 1, a one-way analysis of variance (ANOVA) showed that there was no significant difference in participants’ trait-levels of empathy on any subscale across the four conditions (Perspective Taking: *F*(3, 435) = .89, *p* = .446, *ŋ*^*2*^ = .006, Empathic Concern: *F*(3, 435) = 1.09, *p* = .353, *ŋ*^*2*^ = .007, and Personal Distress: *F*(3,435) = 1.275, *p* = .282, *ŋ*^*2*^ = .009). There was also no significant difference across conditions when it came to beliefs about empathy (Controllability: *F* (3, 435) = .339, *p* = .805, *ŋ*^*2*^ = .002, Implicit Theories: *F* (3,435) = .461, *p* = .709, *ŋ*^*2*^ = .003). The lack of significant differences across conditions for our population variables suggests that all of the conditions were well balanced in terms of individual differences, and that random assignment to the four conditions was successful.

### Outcome variables

#### Manipulation check

Cohen’s Kappa was calculated in order to assess inter-coder reliability and agreement between the two coders [68]. There was substantial agreement between the two coders (κ = .93, *p* < .001, 95% CI [.87, .98]. Data from the two coders were averaged together. A Chi-Squared test was used in order to test whether or not there was a significant difference in the proportion of participants who reported feeling present and the participants who did not between conditions. In the NPT condition 2% of participants received a score of 1, in the Desktop condition 9.2%, and in the VRPT condition 30% of participants received a score of 1. Results showed that significantly more participants in the VRPT condition reported feeling spatially present than participants in the Desktop condition (*X*^*2*^(1) = 13.37, *p* < .001; 95 CI [9.82, 31.30]) and the NPT condition (*X*^*2*^(1) = 28.72, *p* < .001; 95 CI [18.43, 37.67]). Participants in the Desktop condition also reported feeling more spatially present than participants in the NPT condition (*X*^*2*^(1) = 4.78, *p* < .029; 95 CI [.55, 14.73]). These results confirm that our experimental conditions are significantly different from each other in terms of presence with the VRPT condition being the most immersive, followed by the Desktop condition, and then the NPT condition. The Information condition responses were not coded because participants in this condition did not perform any kind of perspective-taking task.

#### Continuous variables

The means and standard deviations for the continuous variables (i.e., IOS, Dehumanization, Empathy, Personal Distress, Presence, support for Proposition A, and amount donated) are summarized in Table 5. A one-way, between-subjects analysis of variance (ANOVA) was carried out to compare the effect of condition on all of the continuous outcome variables. All significant effects of condition on the outcome variables were followed up with three planned orthogonal contrasts that specifically tested our hypotheses. We predicted 1) that any type of perspective-taking would be more effective at eliciting empathy and prosocial behaviors than receiving information (i.e., the three perspective-taking conditions vs. Information), 2) that mediated perspective-taking would be more effective than NPT (i.e., Desktop and VRPT vs NPT), and 3) that the most immersive perspective-taking task would be more effective than the less immersive perspective-taking task (i.e., VRPT vs Desktop). A Bonferroni adjusted alpha (*p = α/k)* was utilized in order to account for multiple comparisons and avoid Type I errors.

**Table 5:** Means and standard deviations for all outcome variables by condition in Study 2.

Measures	Information *M (SD)*	NPT *M (SD)*	Desktop *M (SD)*	VRPT *M (SD)*
IOS	2.45 (1.47)	2.89 (1.94)	3.27 (1.68)	3.04 (1.75)
Dehumanization	91.86 (15.17)	87.15 (20.46)	92.22 (12.74)	88.45 (14.82)
Empathy	4.79 (1.32)	5.31 (1.10)	5.21 (1.12)	5.16 (1.26)
Personal Distress	3.78 (1.47)	4.29 (1.41)	4.10 (1.34)	4.37 (1.45)
Presence	-	-	2.78 (.81)	2.98 (.82)
Support for Prop A	3.91 (.84)	3.75 (1.03)	3.89 (.97)	4.18 (.79)
Amount Donated	7.63 (3.69)	7.86 (3.61)	8.2 (3.28)	8 (3.42)
Petition Signing proportion	80 out 107	66 out of 104	75 out of 113	98 out of 115

*M* = Mean; *SD* = Standard Deviation.

IOS. There was a significant effect of condition on self-other overlap with the homeless: *F* (3, 435) = 7.197, *p* = < .001, *ŋ*^*2*^ = .05. There was a substantial difference in self-other overlap between the Information and the three perspective-taking conditions (*F* (1, 435) = 18.97, *p <* .001). Participants felt closer and more connected to the homeless after any type of perspective-taking than when they just received information about the homeless. This result replicates extant research highlighting the effectiveness of perspective-taking tasks on self-other overlap [73]. However, there was no significant difference between the NPT and the mediated perspective-taking conditions (*F* (1, 435) = 1.66, *p* = .197) or the mediated conditions themselves (*F* (1, 435) = .96, *p* = .328). These results replicate the findings from Study 1, highlighting that mediated perspective-taking tasks, regardless of how immersive they are, do not result in participants feeling more connected to the homeless than traditional perspective-taking tasks.

Taken together, these results showed that just receiving information about the current state of the homeless population did not result in participants feeling closer or more connected to the homeless population. However, perspective-taking tasks, regardless of delivery medium or level of immersion, had a positive effect in that participants felt more connected to the homeless after taking their perspective.

Dehumanization. The dehumanization scale measured how evolved participants felt the average member of the homeless population was at the time of the study. There was a significant effect of condition on dehumanization: *F* (3, 435) = 2.68, *p* = .046, *ŋ*^*2*^ = .02). However, there was no significant difference in how evolved participants reported the homeless to be between the Information and the three perspective-taking conditions (*F* (1, 435) = 2.10, *p* = .147), only a marginally significant difference between the NPT and mediated perspective-taking conditions (*F* (1, 435) = 2.85, *p* = .092) indicating that mediated perspective-taking led to higher (less dehumanizing) scores than NPT. There was also a marginally significant difference between the Desktop vs VRPT conditions (*F* (1, 435) = 3.09, *p* = .079) where participants in the less immersive perspective-taking task thought of the homeless as more evolved than the participants in the VRPT condition.

Empathy. There was a significant effect of condition on self-reported empathy: *F* (3, 435) = 3.14, *p* = .025, *ŋ*^*2*^ = .02. Participants in the three perspective-taking conditions reported feeling significantly more empathy toward the homeless than participants in the Information condition (*F* (1, 435) = 9.31, *p* = .002). However, there were no significant differences between the two types of mediated perspective-taking tasks (*F* (1, 435) = 0.032, *p* = .858) or between them and the NPT condition (*F* (1, 435) = 0.063, *p* = .802). Similar to the self-other overlap results, there was no difference between the participants in the NPT condition and the two mediated conditions (Desktop and VRPT).

Personal Distress. There was a significant effect of condition on how distressed participants reported feeling after the intervention: *F* (3, 435) = 3.743, *p* = .011, *ŋ*^*2*^ = .03. There was a significant difference in personal distress between the Information and the three perspective-taking conditions (*F* (1, 435) = 8.93, *p* = .003) with participants in the Information condition reporting less distress than their counterparts. As with the previous analyses of the self-other overlap and empathy scales, there was no significant difference in reported personal distress between the mediated conditions (*F* (1, 435) = 0.28, *p* = .595) or between them and the NPT condition (*F* (1, 435) = 2.01, *p* = .156).

These results show that participants in the three perspective-taking conditions reported feeling more empathetic and connected to the homeless than participants who only received information about the current state of homeless population. Participants in the three perspective-taking conditions also reported feeling more personally distressed after taking the perspective of a homeless person than participants in the Information condition.

Social Presence. Social presence, or the feeling of being inside a virtual environment with others was only measured for the Desktop and VRPT conditions. There was only a marginally significant difference (*t*(229) = 1.82, *p* = .070; 95 CI [-0.4, .01]), with the Desktop condition (*M* = 2.78, *SD* = .81) being lower than the VRPT condition (*M* = 2.98, *SD* = .82).

Replication of Study 1. Study 1 compared NPT and VRPT immediately after the intervention. However, Study 2 does not directly compare NPT and VRPT, but rather compares NPT to both VRPT and Desktop conditions, making it unclear whether or not some of the findings in Study 1 were replicated in Study 2. In order to be able to compare the IOS, dehumanization, empathy, and personal distress findings from Study 2 with those of Study 1 the NPT and VRPT conditions were compared to each other.

In Study 2, there were no significant differences in IOS scores between the NPT and VRPT condition (*t* (211) = -1.59, *p* = .554; 95 CI [-0.64, 0.34]). There were also no significant differences immediately after the perspective-taking tasks in terms of dehumanization scores (*t*(191) = -0.53, *p* = .594; 95 CI [-6.10, 3.50]). Both of these results replicate the findings from Study 1. There were also no significant differences in empathy (*t*(215) = -0.24, *p* = .812; 95 CI [-6.10, 3.50]) or personal distress (*t*(191) = -0.53, *p* = .594; 95 CI [-6.10, 3.50]) between the NPT and VRPT conditions; this pattern deviates from Study 1 where there were significant differences between NPT and VRPT immediately after the intervention.

Correlations among Dependent Variables. All simple correlations are included in Table 6. A Holm-Bonferroni correction was implemented to account for multiple comparisons. Results show the adjusted p-values. The correlation analysis showed that IOS scores were significantly and positively correlated with self-reported empathy (r = .15, *p* = .025), and social presence (r = .20, *p* = .049). In other words, participants who reported feeling more connected to the homeless also reported more empathy, and felt more copresent with virtual humans in the mediated perspective-taking tasks (i.e., Desktop and VRPT). Self-reported empathy was significantly and positively correlated with personal distress (r = .39, *p* < .001) and social presence (r = .23, *p* = .006).

**Table 6:** Simple correlations among dependent variables for Study 2.

	Dehumanization	Empathy	Personal Distress	Social Presence	Support Prop A	Donation
IOS	0.13	**0.15** ^a^	0.07	**0.20** ^a^	0.08	0.01
Dehumanization		0.06	-0.03	-0.06	0.03	-0.04
State Empathy			**0.39** ^c^	**0.23** ^c^	0.03	0.04
Personal Distress				0.15	0.07	0.07
Social Presence					-0.02	-0.08
Support Prop A						0.11
Donation						

^a^ positive at p < .05.

^b^ positive at p < .01.

^c^ positive at p < .001.

#### Behavioral measures

Support for Proposition A. There was a significant effect of condition on support for Proposition A: *F* (3, 435) = 4.29, *p* = .005, *ŋ*^*2*^ = .03. There was no significant difference between the Information condition and the three perspective-taking conditions (*F* (1, 435) = 0.107, *p* = 0.744*)*. However, participants in the mediated perspective-taking conditions (i.e., Desktop and VRPT) supported Proposition A significantly more than participants in the NPT condition (*F* (1, 435) = 7.99, *p* = .005). There was also a significant difference between the VRPT and Desktop conditions (*F* (1, 435) = 4.77, *p* = .029) where participants in the VRPT condition reported supporting Proposition A significantly more than participants in the Desktop condition.

Signing Petition supporting Proposition A. There was a significant effect of condition on the proportion of petitions signed (Fisher’s exact test: *p* = < .001). The first planned contrast (Information vs all perspective-taking conditions) showed that there was no significant effect of perspective-taking on petitions signed (*z* = 0.36, *p* = .722). However, the second planned contrast between mediated and traditional perspective-taking tasks showed that a significantly higher proportion of participants in the Desktop and VRPT conditions signed the petition in comparison to the participants in the NPT condition (*z* = -2.53, *p* = .0113). Finally, significantly more participants in the VRPT condition signed the petition than participants in the Desktop condition (*z* = -3.25, *p* = .001).

These results show that mediated perspective-taking, regardless of immersion level, is more effective at encouraging political action in the form of signed petitions than NPT. However, increased immersion leads to significantly more signed petitions (significantly more participants in VRPT condition signed the petition than participants in the Desktop condition).

Donation Question. When it comes to the amount of money donated to a homeless shelter, there was no significant effect across conditions: *F* (3,435) = .528, *p* = .663, *ŋ*^*2*^ = .004. On average, participants in all conditions donated between $7 and $8 dollars to a homeless shelter, with 70% of all participants (*n* = 310) donating the maximum amount possible ($10). Since such a large percentage of participants chose the highest possible amount, this measure was at ceiling, and did not let us accurately conclude whether or not a specific type of empathy intervention was more effective at promoting prosocial behaviors in the form of donations than the others.

Replication of Study 1. In order to be able to compare the behavioral measures from Study 2 with those of Study 1, the NPT and VRPT conditions were compared to each other. Results showed that there was a significant difference in self-reported support for Proposition A (*t*(217) = 3.53, *p* < .001; 95 CI [.19, .68]). However, there was no significant difference in support for Proposition A in Study 1. When it comes to donations to a homeless shelter, there were no significant differences in amount donated between the NPT and VRPT participants (*t*(217) = 0.57, *p* = .567; 95 CI [-0.66, 1.21]), replicating the results from Study 1. Also replicating the results of Study 1, there was a significantly higher proportion of participants in the VRPT condition that signed the petition supporting affordable housing than participants in the NPT condition (*t*(218) = 3.61, *p* < .001; 95 CI [0.56, 1.87]).

## Discussion

Overall, the self-report results show that the Information condition, which solely provided facts about the homeless population, was less effective at making participants feel empathetic and connected to the homeless than any of the perspective-taking conditions. Participants in the Information condition also reported feeling significantly less distressed and rated the homeless as less evolved than any of the perspective-taking participants. However, there was no significant difference between the Information condition and the VRPT condition in the proportion of people who signed the petition. These results show that information interventions can help promote prosocial behaviors, however, it is unclear whether empathy, social desirability, or increased awareness motivated these behaviors.

Showing the opposite pattern, participants in the NPT and Desktop condition reported feeling as empathetic and connected to the homeless as the participants in the VRPT condition. However, a smaller proportion of participants in the NPT and Desktop conditions signed the petition in support of Proposition A than participants in the VRPT condition. The immersive experience of becoming homeless in an IVE resulted in a significantly higher proportion of participants exhibiting helpful behaviors toward the homeless in the form of signing a petition when compared to traditional and less immersive perspective-taking tasks.

## General discussion

Across two studies, we compared the effect of VR perspective-taking tasks against more traditional and less immersive perspective-taking tasks. We hypothesized that the more immersive the perspective-taking task, the more empathy and prosocial behaviors participants would exhibit toward the homeless.

Study 1 was a longitudinal investigation that compared the effects of a VR perspective-taking task against a more traditional, narrative-based perspective-taking task at the time of the intervention and over the course of eight weeks. Results showed that there was no significant difference in self-other overlap (i.e., the extent to which participants felt connected to the homeless) at the time of the intervention or over the course of eight weeks. At the time of the intervention, participants in the VRPT condition reported feeling more empathy toward the homeless and more personal distress than participants in the NPT condition. However, over the course of the eight weeks after the intervention, these differences dissipated and there was no significant difference between conditions in self-reported empathy or personal distress. Given we employed an imagine-self perspective taking task, the empathy and personal distress results were expected, and replicate Batson, Early, and Salvarani’s [17] results. These findings add to the literature by providing empirical evidence showing that imagine-self perspective-taking tasks, regardless of delivery medium, result in a combination of other-oriented empathy and self-oriented distress.

Unlike reported empathy and personal distress, there were no significant differences between conditions in blatant dehumanization or attitudes toward the homeless immediately after the intervention. Over time, however, participants in the VRPT condition had more positive attitudes and thought of the homeless as more evolved than participants in the NPT condition. Even though there was no significant difference of blatant dehumanization at the time of the intervention, these results show that the positive effect of traditional perspective-taking on perceptions of the homeless (i.e., lack of dehumanization and better attitudes) is prolonged with the use of VR. These results are consistent with past research showing that attitudes toward a specific social target increase significantly over time even as empathic feelings dissipate [3,71].

Participants in the VRPT and NPT conditions reported similar rates of support for Proposition A immediately after their respective perspective-taking tasks. However, a significantly higher proportion of participants in the VRPT condition signed the petition supporting Proposition A. These results are consistent to those of Rosenberg, Baughman, and Bailenson [55], who saw no significant difference in intention to help but saw significant differences in actual helping behaviors.

Two weeks after the intervention, participants were asked to write a letter to their elected officials about the issue of homeless. Participants in the VRPT condition used significantly more first-person plural pronouns (e.g., we, our, us). Sample statements include “We must find ways to address the reasons why people become and stay homeless: job loss, mental health issues, high cost of living, lack of affordable housing” and “We must strive to be a community [that] reaches out to support all of us, and we must think about ways to support those of us who currently do not have a place to stay.” Another VRPT participant wrote “Homelessness is a pressing issue in our community, and we have clear moral and civic obligations to take action to help people in need.” When writing to their elected officials about the issue of homelessness and its possible solutions, participants in the VRPT condition included themselves as part of the solution rather than telling the elected official what they or the government should do.

Four weeks after the intervention, participants were asked the extent to which they agreed with Measure B, a measure that advocated for affordable housing just like Proposition A. Participants in the VRPT condition reported more support for Measure B than NPT participants. These results showed that over time, participants in the VRPT condition continued to support political initiatives that could actually benefit the homeless population, whereas the level of support for these kinds of initiatives decreased significantly over time for the participants in the NPT condition.

Study 2 expanded on Study 1 by further exploring the mechanisms that caused differences between the narrative-based perspective-taking and the VR perspective-taking task. We compared the effect of three different types of perspective-taking tasks, each varying in levels of immersion, against each other and against a fact-driven information intervention in order to more accurately explore the effect of immersion and type of empathy intervention on elicited empathy and prosocial behaviors toward the homeless. Study 2 also differed from Study 1 in that we used a larger, more racially diverse sample with participants ranging from 15 to 88 years old. In line with our predictions, and replicating past studies [3], the results of Study 2 showed that participants in all three perspective-taking conditions reported feeling more empathetic toward the homeless compared to participants in the Information condition who did not perform a perspective-taking task at all.

When comparing the three different types of perspective-taking tasks, it was participants in the VRPT condition who reported feeling more connected and empathetic toward the homeless than the less immersive Desktop condition and the NPT condition. Replicating results of Study 1, a significantly higher proportion of participants in the VRPT condition signed the petition supporting efforts to increase affordable housing than participants in the Desktop or NPT conditions.

On most outcome variables (i.e., self-other overlap, reported empathy, and personal distress) participants in the information condition reported feeling less connected and less empathetic toward the homeless than participants in the three perspective-taking conditions. At first glance, these results were expected since past research has shown that giving people information does not always change their attitudes [29,74]. When it comes to petition signatures, more participants in the VRPT condition signed the petition than participants in the Information condition. However, this difference was not statistically significant. These results show that fact-driven interventions can also be successful at promoting prosocial behaviors. Additionally, the discrepancy in self-reported empathy results and signed petitions for participants in the Information condition suggests that empathy or self-other overlap were not the only mechanisms that led to prosocial behaviors.

In terms of dehumanization, there was no significant difference between the Information condition and the PT conditions. However, there was a significant difference between the immersive conditions and the NPT condition. This result was expected since we hypothesized that higher levels of immersion would lead to more empathy and more positive attitudes. There was also a marginally significant difference between the Desktop and the VRPT conditions, with Desktop participants evaluating the homeless as more evolved than the VRPT participants immediately after the intervention. This result was unexpected, but could potentially be explained by the fact that participants in the VRPT condition viscerally experienced harassment in one of the bus scenes from a fellow rider. This experience may have influenced how participants thought of the homeless since they were personally accosted by someone inside the VR experience. While participants in the Desktop condition went through the exact same scene, they were not immersed in the environment, and their personal space was not violated. However, these results did not negatively affect prosocial behaviors. A significantly higher proportion of participants in the VRPT condition signed the petition in support of Proposition A than participants in the Desktop condition.

The dehumanization score results in Study 2 may be an example of how depicting a real scenario, such as being accosted by someone inside an IVE, can have undesirable or unintended effects when compared to depicting the same scenario through a less immersive medium (e.g., desktop computer or video). This concern becomes particularly salient as consumer adoption of VR systems continues to increase and empathy-driven VR experiences become more available to the public. Past research has demonstrated that short VR experiences can have visceral reactions that affect the way a person thinks, feels, and behaves [38, 53, 55]. There is also cogent evidence that virtual humans, such as the man in the bus scene, exert social influence over users [75]. In general, as VR scales up and more people have access to it, it is important for designers and researchers to pilot test their experiences to ensure the experiences they create are having the intended effect.

Study 2 replicated the IOS and dehumanization scale findings from Study 1. In both studies, there was no significant difference in IOS and dehumanization scores between the NPT and VRPT conditions. The results of both of these studies provide more evidence suggesting that when VR perspective-taking tasks are employed, valuations of the social target do not increase significantly at the time of the intervention, but tend to increase over time, and last up to two months after the intervention. However, when comparing the empathy and personal distress results, Study 2 did not replicate the findings from Study 1. Study 1 found that participants in the VRPT condition reported more empathy and personal distress immediately after the intervention, but Study 2 found no significant differences between these two conditions. One of the likely reasons the empathy and personal distress findings from Study 1 were not replicated is that the sample used in Study 2 was much larger and more demographically diverse than the sample used in Study 1. The Study 2 sample was also mostly composed of volunteers that had never used a VR headset before. It is possible that the novelty of the equipment acted as a distraction and prevented participants from focusing on the experience itself, resulting in lower empathy and personal distress scores.

In a longitudinal study, Bailenson and Yee [76] found that, over time, VR users change the way they behave inside virtual environments and suggest that the novelty of VR technology has an effect on virtual social interactions. They also propose that the way experienced VR users go through a VR experience and use the technology is different from the way that first-time users experience the technology [76]. As VR empathy-driven interventions begin to be used at scale, it will be important to control for the level of experience of the users. However, at this time, more research is needed in order to assess the full extent to which the benefits of VRPT are moderated by the participant’s level of experience with VR technology.

Overall, these findings replicate past VR studies in which participants who embodied the perspectives of other groups (e.g., colorblind individuals and the elderly) performed more helping behaviors than participants who did not use VR [5, 40]. Results from the present investigation further confirm that VR, compared to other types of traditional or mediated perspective-taking, often leads to better attitudes and a higher proportion of users signing petitions in support of helpful initiatives for outgroup members.

### Limitations and future directions

There are a number of limitations to these studies. First, the manipulation check assessing the level of spatial presence felt by participants between the different conditions in Study 1 and Study 2 was derived from participants’ written responses to an open-ended question instead of a quantitative, self-reported measure of spatial presence. Additionally, this investigation only included a quantitative measure of social presence. In future studies, participants will be explicitly asked to rate the extent to which they felt spatially present, socially present, and self-present to better understand how these different dimensions of presence impact empathic and behavioral outcomes after VR perspective taking tasks.

Second, in Study 2, participants in all four conditions donated money to a homeless shelter at similar rates (between $7 and $8 dollars). Even though participants in the VRPT condition donated more money than the rest of the conditions, the donation question was at ceiling, meaning more than 70% of all participants across the four conditions chose to donate the maximum amount possible. The donation question was flawed in that it did not give a wide enough range of possible answers in order to address the possible differences between conditions or between different types of prosocial behaviors (i.e., signatures for Proposition A). Participants were also compensated with $10 dollars for their participation, therefore it is likely that the full burden of donating money was not felt by some of the participants. Even though on average most participants donated around the same amount of money, the standard deviations were high for each condition. Such high variability in amount donated across conditions prevented the researchers from being able to examine whether or not different types of perspective-taking tasks led to different levels of prosocial behaviors. Future studies should ask participants how much money out of their own pocket they would like to donate or allow for a wider range of possible answers (e.g., $0 to $100) in order to address these limitations.

It is important to note that the VR experience used immersed participants in an environment specifically designed to provide a visceral experience of what it would be like to be homeless from the first person perspective. However, the VR experience lasted approximately 15 minutes and was not able to simulate some of the psychological and physiological burdens that homeless people experience (e.g., desperation or hunger). These limitations prevent participants from actually experiencing what it would be like to become homeless. Another limitation of the technology is that despite the high level of interactivity within the experience, it still did not allow to participants to interact with the virtual world the way they naturally interact with the real world.

Another limitation of these studies is that attitudes toward the homeless were not measured before the intervention. Even though the participants were randomly selected into each condition, it is possible that participants already had set views regarding the homeless that the researchers were not aware of. Future studies should measure pre-existing biases and attitudes toward the homeless in order to more accurately assess the effect of the different types of empathy interventions. Additionally, the design of the present investigation lacked a pure control condition. In Study 2, the Information condition led to an unexpected number of donations and proportion of signatures supporting affordable housing. A pure control condition, without any kind of empathy intervention, would be necessary in order to address the mechanism that led to these prosocial behaviors.

Results of Study 2 found that a similar proportion of participants in the VRPT and the Information conditions signed the petition supporting affordable housing for vulnerable populations. Given participants in any of the perspective-conditions reported feeling more connected, more empathetic, and more personally distressed than participants in the Information condition, it is unlikely that empathy led to these behavioral outcomes. It is possible that social desirability or increased awareness about the homeless led to these results. Future studies should compare a pure control condition, an information condition, and a perspective-taking task condition to try to understand the mechanism that makes information-driven interventions successful at promoting prosocial behaviors.

Despite these limitations, the results of this investigation provide encouraging evidence supporting the use of IVEs to promote empathy and prosocial behaviors toward extreme outgroup members. However, it is important to note the small effect sizes, and the fact that for most of the self-report measures in Study 2 there were no significant differences between the three perspective-taking conditions. This might be due to the fact that the content was as rigorous in all of these conditions, and that the level of immersion did not have as much of an effect eliciting more empathy. Future studies should try to replicate these results while targeting a different social group in order to assess the generalizability of VR as a perspective-taking tool, and test whether it is the content and context that allows perspective-taking to promote empathy or rather the modality of the medium by which the intervention is administered. Future studies should also consider using implicit measures of empathy in addition to explicit self-report measures in order to further understand the patterns exhibited between self-report and behavioral measures.

In the current version of the VR experience, participants are able to make choices about what they want to sell, what search strategy they implement in the car, how they react and respond to the men in the bus, and who and how they interact with the other homeless people in the bus. Participants are constantly seeing how their decisions affect the narrative and this, in turn, provides a highly individualized and interactive experience for the participant. Future studies should compare this type of VR experience to one in which participants do not have agency (i.e., a VR experience without interactivity) in order to assess the role that interactivity plays in self-reported empathy, attitudes toward a specific social target, and prosocial behaviors. We speculate that highly interactive and responsive IVEs, where participants are able to see how their own actions manifest in the IVE in real-time, may lead to higher levels of spatial and social presence, more self-reported empathy, better attitudes, and more prosocial behaviors. However, whether or not this difference is significant requires more empirical evidence.

Moreover, future research should compare imagine-self and imagine-other VR perspective-taking tasks. The present study employed imagine-self perspective-taking tasks and found some effects on behavioral measures, however, the motivation that led to these behaviors is not clear. Past research suggests that imagine-self tasks evoke both other-oriented empathy and self-oriented distress, and that this combination of emotions can lead to a stronger motivation to help when compared to imagine-other tasks [17]. Thus, it would be expected that more helping behaviors would be performed with an imagine-self task than an imagine-other task. A study comparing these two types of perspective-taking would be able to discern whether the motivation to help was altruistic or egoistic, and compare the amount of prosocial behaviors performed by participants.

Finally, future research should examine the effect of novelty on experimental outcomes. Past research has demonstrated that users change the way they behave inside virtual environments once they have been exposed to the technology a number of times [76]. These results suggest that the level of experience or familiarity with VR technology may have an effect on the way users interact with each other and in the way that they experience VR in general. However, more research is needed in order to assess the impact of novelty on empathy-driven VR experiences and interventions. Future studies should control for the number of times participants have used VR technology to see if novelty moderates elicited empathy and prosocial behaviors after a VR perspective-taking task.

## Conclusion

The present investigation found that over the course of eight weeks, participants who completed a VR perspective-taking task had more positive attitudes and signed a petition supporting helpful initiatives toward the homeless at significantly higher rates than the participants who just imagined what it would be like to become homeless or performed a less immersive perspective-taking task. The investigation also found that narrative-based and mediated perspective-taking interventions, regardless of immersion level, are more effective at increasing self-reported empathy than interventions without any perspective-taking tasks. The results of this investigation provide evidence suggesting that VR perspective-taking tasks may be more effective at improving attitudes toward specific social targets and motivating prosocial behaviors in the form of signed petitions in support of helpful initiatives than traditional and less immersive perspective-taking tasks.

## Supporting information

S1 Appendix Materials. Contains all of the referenced texts.
(DOCX)

S2 Appendix Questionnaires. Contains all of the questionnaires used in Study 1 and Study 2.
(DOCX)

S1 File Additional analyses. Contains additional analysis examining social presence as a moderator.
(DOCX)

S1 Dataset Complete dataset for Study 1. Contains all of the raw data and open-ended responses used for analysis in Study 1.
(XLSX)

S2 Dataset Complete dataset for Study 2. Contains all of the raw data and open-ended responses used for analysis in Study 2.
(XLSX)
