# Supplementary material for: Building long-term empathy: A large-scale comparison of traditional and virtual reality perspective-taking
Source: PLoS One. 2018 Oct 17;13(10):e0204494. doi: 10.1371/journal.pone.0204494 (PMC6192572; doi:10.1371/journal.pone.0204494)
Supplement: S1 Appendix — Contains all of the referenced texts. (DOCX) [file pone.0204494.s001.docx]

**BUILDING LONG-TERM EMPATHY: APPENDIX**

**Text A: NPT Narrative**

You finally get home and turn on the radio that is sitting on top of your bookshelf. You hear the following report:

*“[Welcome back] to the show, this is Derek with your daily news dish. While the US unemployment rate has hit a post-recession low, industry in this area has had to deal with significant layoffs this year. Layoff plans are either in the works or have already been implemented for a number of companies. With technology in this area advancing rapidly, companies are trying to eke out profits to keep up with large companies by downsizing, refocusing, and restructuring. It’ll be interesting to see what profits these companies turn out next quarter, but investors are quite happy with these decisions overall…”*

You look down at the table and you see newspaper clippings with job advertisements you have circled. You hear a multiple knocks on the door.

*“Hey! This is your landlord...I know you’re in there. You really need to pay me for the last two month’s rent. If you can’t get me at least $700 in the next 12 days I’m going to have to evict you.”*

Seven days pass and just like he said, there is an eviction notice on your door.

Not having a job for the last couple months has been difficult. You owe $700, but you can only afford to pay $200 at this time. To make up the difference, you decide to start selling your belongings.

It doesn’t matter how much you sell, you still can’t raise the $700. You hear your landlord at the door again.

*“This is your landlord, open up! You’re gonna have to leave. I’m sorry, but if you can’t pay then you can’t stay here.”*

You no longer have a roof over your head. You decide to stay with a friend for a couple of days but feel like a burden and end up leaving. Your only choice now is to live out of your car.

You are in your car with what is left of your belongings. Your phone lights up letting you know you have a voice mail.

*“Hey, can you call me back when you get a chance? I’m really concerned about you sleeping in your car at night. You know you can stay with me longer, it’s not a burden. Plus I know you’ll get back on your feet soon. Your new job will help. I know they don’t pay as much as you’d like but you could probably move up in the company soon”*

You turn on the radio to clear your mind.

*“Welcome back to KFM 98.2, this is Derek, with your nightly news dish. A police operation launched today that will crack down on the number of homeless people sleeping in their cars at night. The operation was launched in response to concerns raised by local residents and businesses about community aesthetic and safety. The crackdown comes just two weeks after the city council passed an ordinance that calls for the citation of any person or persons found living out of their cars in public places. Those who violate the ordinance multiple times could face vehicle impoundment. I’d like to open this topic up for discussion, cities across the nation have begun taking action against their homeless population in this way, what are your thoughts? Call in and let us know.”*

You are done for the day, it is now time to get ready to go to sleep. You look around the car for your toothbrush so you can brush your teeth. It might be located under some of the other items in the car but you don’t know exactly where. Everything you own is in that car. After looking for a while you finally find your toothbrush, but you can’t find the toothpaste. You move everything around until you find it. You think you’re ready to brush your teeth but since you’ve moved everything around in the car, now you can’t find your water bottle or the plastic cup you use to spit out your toothpaste. You suddenly hear police sirens the red and blue lights flash from behind the car, the sound of footsteps approaches and a flashlight shines into the your car.

*“Wake up, hey! (Knock Knock Knock) do you have a permit to park here?... it doesn’t look like it. It looks like you’ve been living in your car… you know you’re not allowed to do that, okay? I’m sorry, but I’m gonna have to cite you.”*

Because of the citation you can’t live in your car anymore so you tried to stay in a local homeless shelter. Unfortunately, shelters in this area are highly impacted. You’d have to arrive around 4:30pm to have a chance of getting a bed that night. That means you’d have to leave work early, risking losing your job. For many people like yourself, poverty can become a cycle that is nearly impossible to escape.

For the price of a bus ticket, you and other homeless people seek shelter and warmth on this bus, as you ride it from the start of its line to the end of its line every night. This form of shelter only lasts about 3 hours, until the bus reaches the end of the line. At that point, everyone is woken up and must get off the bus while the bus driver takes a 10 minute break. Then everyone gets back on and the cycle continues.

You look at the man sitting in front of you. You’ve seen him on the bus before. Other homeless people say he regularly steals from their bags while they’re sleeping. Despite being off the streets, sleeping on this bus is not easy to do. If you’re able to sleep with the noise and the movement, you must also worry about having your belongings stolen while you sleep. You look back at the man sitting behind you. You notice that he has gotten closer to you. If you don’t regularly check on your belongings, it is possible that they will be stolen, but you also need to keep an eye on the man sitting behind you to make sure he doesn’t get too close to you.

Having to check on your belongings and worrying about your safety makes it really difficult for you to sleep at night.

The bus comes to a jarring stop (imagine a loud braking sound) and blaring overhead lights turn on and there is a voice heard over the loudspeaker telling everyone to get off.

*“Okay! Wake up everybody, end of the line! Wake up, it’s time to go!...”*

Everyone gets off the bus. You gather your belongings and step off the bus. You have nowhere to go.

**Text B: Proposition A Information**

Please read the following information regarding Proposition A.

SAN FRANCISCO AFFORDABLE HOUSING BONDS. To finance the construction, development, acquisition, and preservation of housing affordable to low- and middle-income households through programs that will prioritize vulnerable populations such as San Francisco’s working families, veterans, seniors, disabled persons; to assist in the acquisition, rehabilitation, and preservation of affordable rental apartment buildings to prevent the eviction of long-term residents; to repair and reconstruct dilapidated public housing; to fund a middle-income rental program; and to provide for homeownership down payment assistance opportunities for educators and middle-income households; shall the City and County of San Francisco issue $310 million in general obligation bonds, subject to independent citizen oversight and regular audits?

State law requires that the City’s General Plan describe San Francisco’s housing needs, set goals for providing housing and develop programs to meet those goals. It is City policy to support the construction and rehabilitation of 30,000 new housing units by 2020, with at least 33% of those permanently affordable to low- and moderate-income households, and over 50% within financial reach of middle class households.

The City’s funding for affordable housing comes from property taxes, hotel taxes, developer fees and other local sources. The use of City money triggers the availability of funding from other public and private sources. The City expects that money from these sources will not be enough to meet its future low-, moderate- and middle-income housing goals. The City’s spending of money from general obligation bonds is monitored by the Citizens’ General Obligation Bond Oversight Committee.

The Proposal: Proposition A is an ordinance that would allow the City to borrow up to $310 million by issuing general obligation bonds. The City would use this money to build, buy, improve, and rehabilitate affordable housing in San Francisco.

**Text C: Letter Writing Instructions Given to Participants**

*Letter to an Elected Official*

One of the best ways in which we can help change our community is by communicating with our elected officials.

Elected officials want to know how their constituents feel about issues, especially when those issues involve decisions made by them. Your elected officials usually know what advocacy groups are saying about an issue, but may not understand how a particular decision affects you or those around you.

If you were to write a letter to an elected official, what would it say? Please use the space below to write the first draft of a letter explaining your understanding of the issue of homelessness, stating your position on the issue, and what changes in policy, if any, should take place to make your community a better place.

 Please use the space below to write the letter.

*Letter to a Friend*

Throughout this entire experience you have learned about certain propositions and measures that have been proposed to address the issue of homelessness. You have also imagined or experienced what it is like to become homeless.

In the space below, please write a message to a friend discussing everything you've learned about the issue of homelessness and express your views on the issue. Include what you think should be done, if anything, in order to help homeless people and whether or not the government should use resources to help.

 Please use the space below to write your message.

**Text D: Measure B Information**

Measure B was proposed in Santa Clara County. The purpose of Measure B is:

To provide affordable local housing for vulnerable populations including veterans, seniors, the disabled, low and moderate income individuals or families, foster youth, victims of abuse, the homeless and individuals suffering from mental health or substance abuse illnesses, which housing may include supportive mental health and substance abuse services, shall the County of Santa Clara issue up to $950 million in general obligation bonds to acquire or improve real property subject to independent citizen oversight and regular audits?

This measure passed in Santa Clara County with 67.3% of voters in favor.

**Text E: Information Condition Materials**

Homelessness Statistics – Bay Area 2015

- Estimated homelessness population in 2015 is 6,686.


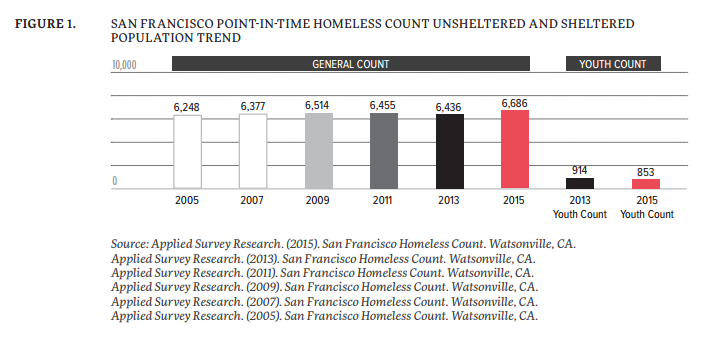


- Homeless people within each district


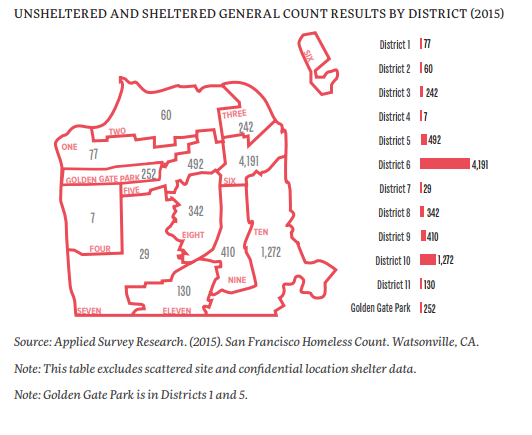


- Persons in families with children represented roughly 9% of the total population counted in the Point-in-Time Count, while roughly 91% were single individuals without families (including unaccompanied children and transitional-age-youth). In total, 6% of those counted on January 29, 2015 were under the age of 18, 20% were between the ages of 18 and 24 and 74% were over the age of 25.
- Based on the 2015 General Count, 57% of the unsheltered homeless population was identified in District 6. The neighborhoods located in District 6 include Tenderloin and South of Market (SoMa). Nineteen percent (19%) of the unsheltered population was identified in District 10, which includes the Bayview neighborhood. While the number of individuals counted in the Bayview decreased between 2013 and 2015, the district represented the second densest population of unsheltered homeless persons in San Francisco on January 29, 2015.
- Eighteen percent (18%) of survey respondents were under the age of 25 at the time of the survey. Twenty-nine percent (29%) were between the ages of 25 and 40, 45% were between age 41 and 60. Eight percent of respondents were 61 years or older at the time of the study.
-
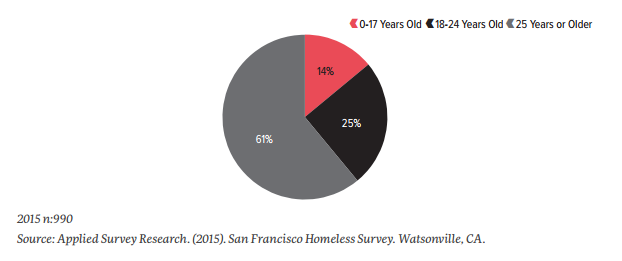
Age at first experience of homelessness:
- In 2015, 21% of respondents reported a history of foster care, higher than in both 2013 and 2011 (18% and 13% respectively). The percentage of youth under the age of 25 who had been in foster care was much higher than adults over the age of 25, 27% compared to 19%.


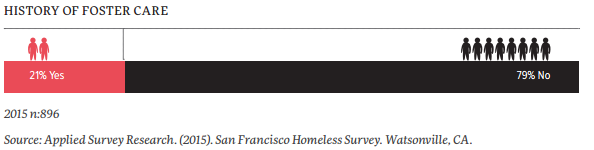

- Nearly half of survey respondents reported currently living outdoors, either on the streets, in parks or encampment areas (46%). Thirty-nine percent (39%) reported staying in a public shelter (emergency shelter, transitional housing facility or alternative shelter environment). Eleven percent (11%) of respondents reported they were sleeping in public buildings, foyers, hallways or other indoor areas not meant for human habitation. Four percent (4%) reported staying in their vehicle.
- For many, the experience of homelessness is part of a long and recurring history of housing instability. Respondents were asked about their current experience or episode of homelessness. More than half of survey respondents (51%) reported they had been homeless for a year or more, a slight decrease from 2013 (54%) but similar to 2011 (51%). One in ten had been homeless for less than one month, slightly higher than in 2013 (8%).
- The primary cause of an individual’s homelessness is not always clear. It is often the result of multiple and compounding causes. One quarter (25%) of respondents reported job loss as the primary cause of their homelessness. Eighteen percent (18%) reported drugs or alcohol, higher than 11% reported in 2013. Twelve percent (12%) reported an argument with a family member who asked them to leave, 11% reported divorce or separation, and 5% reported domestic violence. Thirteen percent (13%) of respondents reported eviction as the primary cause of their homelessness, 3% reported their landlord raised their rent, and 2% reported foreclosure.
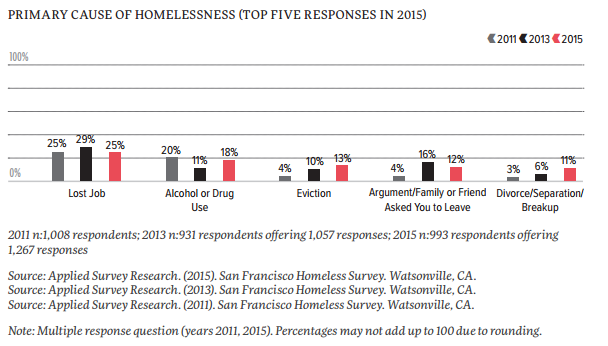

- The greatest percentage (48%) reported they could not afford rent. Twenty-eight percent (28%) reported a lack of job or income. Most other respondents reported a mixture of other income or access related issues, such as the lack of available housing (17%), difficulty with the housing process (13%), or an eviction record (6%). Twelve percent (12%) of respondents reported that a criminal record prevented them from obtaining housing, and 8% reported a medical illness. Eight percent (8%) of respondents reported they did not want housing.
- Nearly three-quarters (72%) of respondents in 2015 reported they were receiving some form of government assistance, up from 54% in 2013. The largest percentage of respondents (40%) reported receiving CalFresh (food stamps) and/or WIC (women, infants, and children food assistance), a slight increase from 2013.
- Of those who reported they were not receiving any form of government support, the greatest percentage reported they did not want assistance (40%). Seventeen percent (17%) did not think they were eligible for services, 13% reported they had never applied, 4% had applied and were waiting for a response, and 5% reported they had been turned down.
- The unemployment rate for homeless respondents was 89%, an increase from 62% in 2013. Eleven percent (11%) of respondents reported working full-time, part-time, or with seasonal, temporary, or casual employment.
- Obstacles for obtaining employment across 5 years: notice needing education/training is no longer an obstacle in 2015


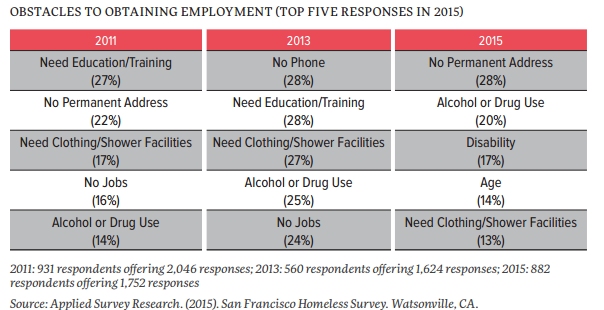


- More than two-thirds of respondents (67%) reported one or more health conditions, higher than in 2013 (63%). These conditions included chronic physical illness, physical disabilities, chronic substance abuse and severe mental health conditions. Thirty-four percent (34%) of survey respondents with these conditions reported their condition limited their ability to take care of personal matters or get or keep a job, similar to 2013.
- The most frequently reported health condition was drug or alcohol abuse (37%), followed by psychiatric or emotional conditions (35%). Twenty-eight percent (28%) reported a physical disability. Seven percent (7%) of respondents reported having AIDS or an HIV related illness.
