# Supplementary material for: Building long-term empathy: A large-scale comparison of traditional and virtual reality perspective-taking
Source: PLoS One. 2018 Oct 17;13(10):e0204494. doi: 10.1371/journal.pone.0204494 (PMC6192572; doi:10.1371/journal.pone.0204494)
Supplement: S2 Appendix — Contains all of the questionnaires used in Study 1 and Study 2. (DOCX) [file pone.0204494.s002.docx]

**BUILDING LONG-TERM EMPATHY: QUESTIONNAIRES USED**

**Questionnaires Used for Study 1 & Study 2**

**Pre-Intervention Questionnaire for both Study 1 and Study 2**

Enter Participant's ID

________________________________________________________________

How old are you?

________________________________________________________________

What gender do you identify as?

- Male
- Female
- Other

**IRI Scale**

The following statements inquire about your thoughts and feelings in a variety of situations.  For each item, indicate how well it describes you by choosing the appropriate letter on the scale at the top of each question:  A, B, C, D, or E.   READ EACH ITEM CAREFULLY BEFORE RESPONDING.  Answer as honestly as you can.  Thank you. A= does not describe me well E = describes me very well

|  | A | B | C | D | E |
| --- | --- | --- | --- | --- | --- |
| I often have tender, concerned feelings for people less fortunate than me |  |  |  |  |  |
| I sometimes find it difficult to see things from the "other guy's" point of view |  |  |  |  |  |
| Sometimes I don't feel very sorry for other people when they are having problems |  |  |  |  |  |
| In emergency situations, I feel apprehensive and ill-at-ease. |  |  |  |  |  |
| I try to look at everybody's side of a disagreement before I make a decision |  |  |  |  |  |
| When I see someone being taken advantage of, I feel kind of protective towards them |  |  |  |  |  |
| I sometimes feel helpless when I am in the middle of a very emotional situation. |  |  |  |  |  |
| I sometimes try to understand my friends better by imagining how things look from their perspective |  |  |  |  |  |

|  | A | B | C | D | E |
| --- | --- | --- | --- | --- | --- |
| I often have tender, concerned feelings for people less fortunate than me |  |  |  |  |  |
| I sometimes find it difficult to see things from the "other guy's" point of view |  |  |  |  |  |
| Sometimes I don't feel very sorry for other people when they are having problems |  |  |  |  |  |
| In emergency situations, I feel apprehensive and ill-at-ease. |  |  |  |  |  |
| I try to look at everybody's side of a disagreement before I make a decision |  |  |  |  |  |
| When I see someone being taken advantage of, I feel kind of protective towards them |  |  |  |  |  |
| I sometimes feel helpless when I am in the middle of a very emotional situation. |  |  |  |  |  |
| I sometimes try to understand my friends better by imagining how things look from their perspective |  |  |  |  |  |

|  | A | B | C | D | E |
| --- | --- | --- | --- | --- | --- |
| When I see someone get hurt, I tend to remain calm |  |  |  |  |  |
| Other people's misfortunes do not usually disturb me a great deal |  |  |  |  |  |
| If I'm sure I'm right about something, I don't waste much time listening to other people's arguments |  |  |  |  |  |
| Being in a tense emotional situation scares me |  |  |  |  |  |
| When I see someone being treated unfairly, I sometimes don't feel very much pity for them |  |  |  |  |  |
| I am usually pretty effective in dealing with emergencies |  |  |  |  |  |
| I am often quite touched by things that I see happen |  |  |  |  |  |
| I believe that there are two sides to every question and try to look at them both |  |  |  |  |  |

|  | A | B | C | D | E |
| --- | --- | --- | --- | --- | --- |
| I would describe myself as a pretty soft-hearted person |  |  |  |  |  |
| I tend to lose control during emergencies |  |  |  |  |  |
| When I'm upset at someone, I usually try to "put myself in his or her shoes" for a while |  |  |  |  |  |
| When I see someone who badly needs help in an emergency, I go to pieces |  |  |  |  |  |
| Before criticizing somebody, I try to imagine how I would feel if I were in their place |  |  |  |  |  |

**Beliefs about Empathy Scale**

**Using the scale below, please indicate your agreement with each of the following statements. There are no right or wrong answers.**

|  | 1 | 2 | 3 | 4 | 5 | 6 | 7 |
| --- | --- | --- | --- | --- | --- | --- | --- |
| People can adjust the amount of empathy they are feeling in any given situation. |  |  |  |  |  |  |  |
| People can control how much empathy they feel for other people in the moment. |  |  |  |  |  |  |  |
| In any given situation, people have the ability to ”turn up” the amount of empathy they feel for someone. |  |  |  |  |  |  |  |
| In any given situation, people have the ability to “turn down” the amount of empathy they feel for someone. |  |  |  |  |  |  |  |
| When a person feels empathy for someone, they can’t stop feeling empathy, even if they want to stop. |  |  |  |  |  |  |  |
| When a person doesn’t feel empathy for someone, they can’t make themselves feel it, even if they want to feel empathy for that person. |  |  |  |  |  |  |  |

|  | 1 | 2 | 3 | 4 | 5 | 6 | 7 |
| --- | --- | --- | --- | --- | --- | --- | --- |
| A person’s level of empathy is something very basic about them, and it can’t be changed much. |  |  |  |  |  |  |  |
| People can always change how much empathy they generally feel for others. |  |  |  |  |  |  |  |
| People can’t really change how much empathy they tend to feel for others. Some people are very empathetic and some aren’t and they can’t change that much. |  |  |  |  |  |  |  |
| No matter who somebody is, they can always change how empathetic a person they are. |  |  |  |  |  |  |  |
| Whether a person is empathetic or not is deeply ingrained in their personality. It cannot be changed very much. |  |  |  |  |  |  |  |
| Anybody can change how empathetic a person they are. |  |  |  |  |  |  |  |

**Post-Intervention Questionnaire for Study 1 and Study 2**

Thank you for participating in our study! 


Please answer the following questions to the best of your ability.

Please enter Participant ID

________________________________________________________________

**IOS**


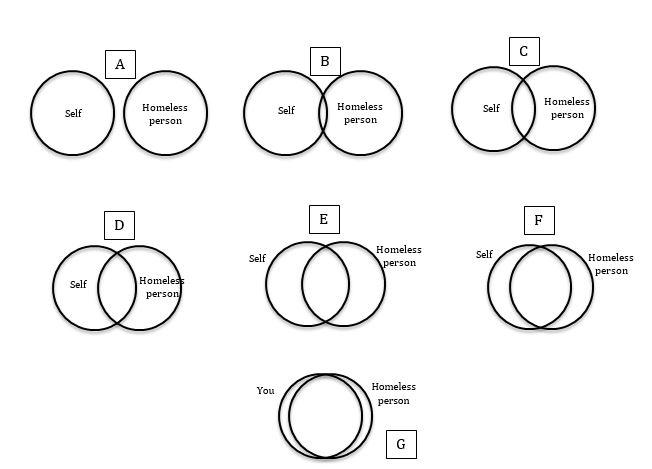


Please indicate which image above best corresponds to the relationship between you and a homeless person.

- A
- B
- C
- D
- E
- F
- G

**Empathy and Personal Distress**

Please indicate the extent to which you experienced the following emotions.

|  | 1 | 2 | 3 | 4 | 5 | 6 | 7 |
| --- | --- | --- | --- | --- | --- | --- | --- |
| Softhearted |  |  |  |  |  |  |  |
| Touched |  |  |  |  |  |  |  |
| Uneasy |  |  |  |  |  |  |  |
| Trouble |  |  |  |  |  |  |  |
| Distressed |  |  |  |  |  |  |  |
| Sympathetic |  |  |  |  |  |  |  |
| Disturbed |  |  |  |  |  |  |  |
| Compassionate |  |  |  |  |  |  |  |

**Attitudes toward the Homeless** (Only in Study 1)

|  | 1 | 2 | 3 | 4 | 5 | 6 | 7 | 8 | 9 |
| --- | --- | --- | --- | --- | --- | --- | --- | --- | --- |
| For most homeless people, it is their own fault that they are homeless. |  |  |  |  |  |  |  |  |  |
| Most homeless people could have avoided becoming homeless. |  |  |  |  |  |  |  |  |  |
| Our society does not do enough to help homeless people. |  |  |  |  |  |  |  |  |  |
| Our society should do more to protect the welfare of homeless people. |  |  |  |  |  |  |  |  |  |

|  | 1 | 2 | 3 | 4 | 5 | 6 | 7 | 8 | 9 |
| --- | --- | --- | --- | --- | --- | --- | --- | --- | --- |
| How much do you personally care about the plight of homeless people? |  |  |  |  |  |  |  |  |  |

|  | 1 | 2 | 2 | 3 | 4 | 5 | 6 | 7 | 8 | 9 |
| --- | --- | --- | --- | --- | --- | --- | --- | --- | --- | --- |
| Compared with other social problems we face today (e.g. crime, education, drugs, AIDS, environmental protection, energy conservation), how would you rate the importance of helping homeless people? |  |  |  |  |  |  |  |  |  |  |

**Dehumanization**
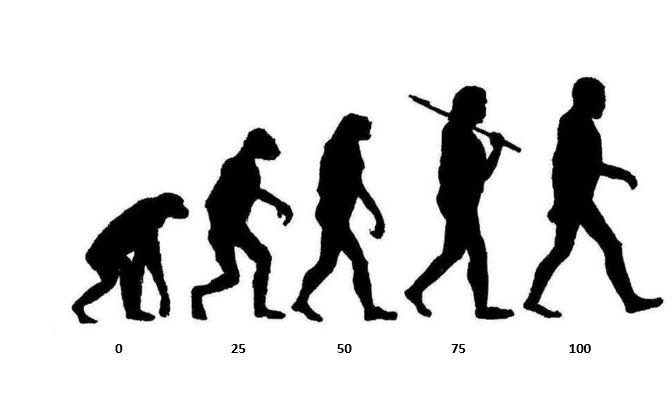


People can vary in how human-like they seem. Some people seem highly evolved whereas others seem no different than lower animals. Using the image below, indicate using the slider how evolved you consider the average member of the group to be.

|  | 0 | 25 | 50 | 75 | 100 |
| --- | --- | --- | --- | --- | --- |

| Homeless People | 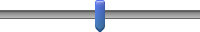 |
| --- | --- |

**Social Presence** (Only for mediated Perspective-Taking tasks)

How strongly did you sense that the homeless people in the bus were present?

- Not at all
- Slightly
- Moderately
- Strongly
- Very Strongly

How strongly did you sense that the homeless people in the bus were watching you?

- Not at all
- Slightly
- Moderately
- Strongly
- Very Strongly

How strongly did you sense that the homeless people in the bus were aware of your presence?

- Not at all
- Slightly
- Moderately
- Strongly
- Very Strongly

How strongly did you sense that the homeless people in the bus were real?

- Not at all
- Slightly
- Moderately
- Strongly
- Very Strongly

How strongly did you sense that the homeless people in the bus were alive?

- Not at all
- Slightly
- Moderately
- Strongly
- Very Strongly

How strongly did you sense that the homeless people were just computer images?

- Not at all
- Slightly
- Moderately
- Strongly
- Very Strongly

**Behavioral Measures Study 1 Time 0 and Study 2**

*Please answer the following questions. Feel free to say yes or no to any of these questions.*

1. To what extent do you support proposition A? Please circle one.

Not at all Completely

1 2 3 4 5

2. Would you be willing to sign a petition to increase affordable housing for homeless people even when it could possibly mean an increase in taxes? If yes, please sign below.

Signature ------------------------------------------------------------------ Date ------------------------------------

*Please answer the following question.*

For participating in this study, you will be compensated with a $10 Amazon gift card. However, you have the opportunity to donate a portion of that compensation to a homeless shelter. For example, if you chose to donate $2 you would receive an $8 gift card. If you chose to donate $4 you would receive a $6 gift card.

Please circle the amount of money that you would like to donate.

0 1 2 3 4 5 6 7 8 9 10

**Behavioral Measures Study 1 Time 1**

One of the best ways in which we can help change our community is by communicating with our elected officials. 
Elected officials want to know how their constituents feel about issues, especially when those issues involve decisions made by them. Your elected officials usually know what advocacy groups are saying about an issue, but may not understand how a particular decision affects you or those around you.
If you were to write a letter to an elected official, what would it say? Please use the space below to write the first draft of a letter explaining your understanding of the issue of homelessness, stating your position on the issue, and what changes in policy, if any, should take place to make your community a better place.  

 Please use the space below to write the letter.

________________________________________________________________

________________________________________________________________

________________________________________________________________

________________________________________________________________

________________________________________________________________

**Behavioral Measures Study 1 Time 2**

To what extent do you agree with Measure B?

- Strongly Disagree
- Slightly Disagree
- Neutral
- Slightly Agree
- Strongly Agree

**Behavioral Measures Study 1 Time 3**

Throughout this entire experience you have learned about certain propositions and measures that have been proposed to address the issue of homelessness. You have also imagined or experienced what it is like to become homeless.

In the space below, please write a message to a friend discussing everything you've learned about the issue of homelessness and express your views on the issue. Include what you think should be done, if anything, in order to help homeless people and whether or not the government should use resources to help.

 Please use the space below to write your message.

________________________________________________________________

________________________________________________________________

________________________________________________________________

________________________________________________________________
