# Supplementary material for: Building long-term empathy: A large-scale comparison of traditional and virtual reality perspective-taking
Source: PLoS One. 2018 Oct 17;13(10):e0204494. doi: 10.1371/journal.pone.0204494 (PMC6192572; doi:10.1371/journal.pone.0204494)
Supplement: S1 File — Contains additional analysis examining social presence as a moderator. (DOCX) [file pone.0204494.s003.docx]

**BUILDING LONG-TERM EMPATHY: ADDITIONAL ANALYSIS**

**Social Presence Interactions.** Additional analyses were carried out to examine whether or not social presence moderated any of the effects found in the self-report and behavioral measures in Study 2. Results show that social presence did not moderate condition effects across any of the continuous outcome variables since all interaction effects were nonsignificant (IOS: *t*(227) *=* -1.18*, p =* .239, 95 CI [-0.87, .22]; Dehumanization: *t*(227) *=* 0.95*, p =* .341, 95 CI [-6.95, 2.42]; Empathy: *t*(227) *=* .079*, p =* .936, 95 CI [-.39, 0.36]; Personal Distress: *t*(227) *=* .91*, p =*.363, 95 CI [-.24, 0.65]). Additionally, social presence did not moderate support for Proposition A (*t*(227)= 0.09*, p =* .518, 95 CI [-0.38, .19]), amount donated to a homeless shelter (*t*(227) *=* 0.10*, p =* .853, 95 CI [-0.99, 1.91]), or petition signatures (*t*(220) *=* 0.18*, p =* .659, 95 CI [-0.62, 1.98]).
